# Lymphocyte-to-high-density lipoprotein ratio and mortality in asthma patients: a novel immunoinflammatory biomarker with nonlinear association

**DOI:** 10.3389/fmed.2025.1553188

**Published:** 2025-06-13

**Authors:** Tu-Lei Tian, Guan-Wei Wu, Mei-Ling Xie, Xiang-Kun Qu, Xiao-Tong Wang, Chang-Lu Sun

**Affiliations:** ^1^Department of Respiratory and Critical Care Medicine, The Affiliated Bozhou Hospital of Anhui Medical University, Bozhou, Anhui, China; ^2^Department of Urology, Affiliated Wuhu Hospital of East China Normal University, Wuhu, Anhui, China; ^3^Bengbu Medical University Graduate School, Bengbu, Anhui, China; ^4^Liaoning University of Traditional Chinese Medicine Xinglin College, Shenyang, Liaoning, China; ^5^Liaoning University of Traditional Chinese Medicine, Shenyang, Liaoning, China

**Keywords:** lymphocyte-to-high-density lipoprotein ratio (LHR), asthma, mortality, competing risk analysis, NHANES

## Abstract

**Background:**

The lymphocyte-to-high-density lipoprotein ratio (LHR), a novel biomarker reflecting systemic inflammation and immune status, has been widely studied in various diseases. However, its association with mortality risk among asthma patients remains unexplored.

**Methods:**

This study utilized data from the National Health and Nutrition Examination Survey (NHANES) spanning 1999–2018, including 5,323 adult asthma patients. Mortality outcomes were ascertained through linkage with the National Death Index (NDI) up to December 31, 2019. Cox proportional hazards models and Fine-Gray competing risk models were employed to examine the association between LHR and mortality risks. Dose–response relationships were assessed using restricted cubic spline analyses.

**Results:**

Over a mean follow-up period of 106.95 months, 724 all-cause deaths (13.6%) were recorded. After multivariable adjustment, a one-unit increase in log-transformed LHR was associated with reduced risks of mortality: 18% for all-cause (HR = 0.82, 95% CI: 0.74–0.91), 21% for cardiovascular disease (CVD) (HR = 0.79, 95% CI: 0.65–0.96), and 41% for chronic lower respiratory disease (CLRD) (HR = 0.59, 95% CI: 0.45–0.77). Restricted cubic spline analyses showed an L-shaped association of LHR with all-cause and CLRD mortality, with inflection points at 1.78 and 1.52, respectively. For CVD mortality, a linear association was observed. Competing risk models further confirmed the association of LHR with reduced CLRD mortality (SHR = 0.64, 95% CI: 0.46–0.88), while the association with CVD mortality was no longer significant (SHR = 0.85, 95% CI: 0.70–1.03).

**Conclusion:**

LHR is nonlinearly associated with all-cause and CLRD mortality and shows a significant inverse association with CLRD mortality risk. These findings were further validated using competing risk models, highlighting the robustness of the results.

## Introduction

Asthma is a chronic inflammatory airway disease characterized by recurrent symptoms such as wheezing, chest tightness, and coughing. According to the World Health Organization (WHO), asthma affects over 340 million people globally, with its prevalence continuing to rise (1). Despite advancements in treatment, approximately 460,000 deaths occur annually due to asthma and its complications (2). Studies have shown that asthma patients face not only an increased risk of mortality from the primary disease but also significantly higher risks from comorbidities such as cardiovascular diseases (CVD), representing a major public health challenge (3, 4). However, the absence of reliable biomarkers to assess inflammation and immune function in clinical practice underscores the urgent need for novel predictive markers to improve risk stratification and optimize clinical management for asthma patients.

Several established biomarkers, including fractional exhaled nitric oxide (FeNO), immunoglobulin E (IgE), and eosinophil counts (both in blood and sputum), are widely used to assess airway inflammation and guide asthma management (5). However, these markers primarily capture local airway inflammation and may not fully reflect systemic inflammation or immune dysregulation, both of which are critical for understanding disease progression and mortality risks (6). Therefore, rather than an absence of reliable biomarkers, there is a pressing need for novel markers that can complement current approaches by integrating systemic inflammation and immune function, thereby improving risk stratification and optimizing clinical management for asthma patients (7).

Emerging evidence highlights the pivotal role of inflammation and immune responses in asthma-related mortality (8). Persistent airway inflammation and immune dysregulation not only accelerate disease progression but also heighten the risk of comorbidities, such as cardiovascular complications, through multiple mechanisms (9, 10). The lymphocyte-to-high-density lipoprotein ratio (LHR) has recently been identified as a novel biomarker of systemic inflammation and immune status. With its simplicity, accessibility, and reliable results, LHR has demonstrated significant prognostic value in various chronic diseases (11). Elevated LHR levels have been associated with adverse cardiovascular outcomes (12) and have shown robust predictive utility in chronic inflammatory diseases such as diabetes and coronary artery disease (13–15). In respiratory diseases, particularly chronic obstructive pulmonary disease (COPD), Huang et al. (16) reported a strong correlation between higher LHR levels and impaired lung function, indicating poorer disease outcomes. However, the relationship between LHR and mortality risk in asthma patients remains unexplored. Given asthma’s inherently chronic inflammatory nature, investigating the association between LHR and mortality risk using large-scale population data from NHANES holds significant clinical relevance.

Building on this background, the present study utilized data from the National Health and Nutrition Examination Survey (NHANES, 1999–2018) cohort of asthma patients, linked to the National Death Index (NDI) database with follow-up through December 31, 2019. This study aimed to evaluate the association between LHR levels and all-cause mortality, CVD mortality, and chronic lower respiratory disease (CLRD) mortality. We further examined the dose–response relationships and tested the robustness of our findings using competing risk models. Considering that asthma patients are at risk of multiple competing events—such as cardiovascular or other respiratory-related deaths—traditional survival analysis methods might overestimate the risk of the primary outcome. By applying the Fine-Gray competing risk model, we can more precisely estimate the cumulative incidence of cause-specific mortality, effectively accounting for the influence of alternative events.

### Study design and population

This study utilized data from NHANES, a publicly available dataset, conducted in the United States between 1999 and 2018. NHANES, overseen by the National Center for Health Statistics (NCHS) under the Centers for Disease Control and Prevention (CDC), employs a nationally representative, stratified, multistage probability sampling design to collect health and nutrition data from the noninstitutionalized civilian population in the United States (17). Information on demographics, socioeconomic status, health behaviors, and health conditions was gathered using standardized questionnaires administered by professionally trained interviewers at recruitment. Physical measurements and laboratory tests were performed by trained medical personnel at mobile examination centers (MECs), following strict standardized protocols (18). This study adhered to the Strengthening the Reporting of Observational Studies in Epidemiology (STROBE) guidelines (19). The NHANES study protocol was approved by the NCHS Ethics Review Board, and participants provided written informed consent before participation (20). No financial compensation or incentives were provided to participants in this study.

**Figure 1:**
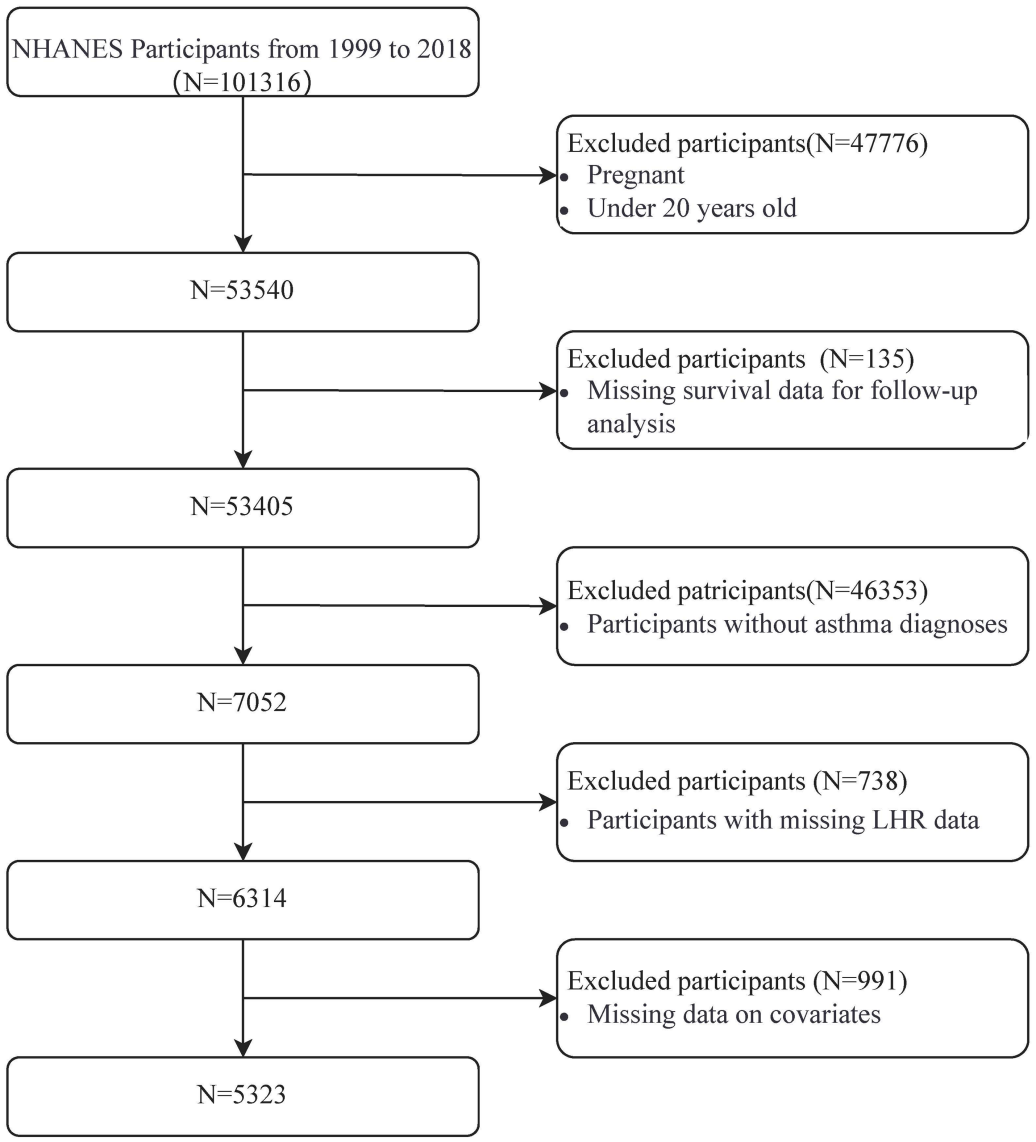
Flow chart of study participants.

A total of 101,316 participants were initially identified from the NHANES dataset (1999–2018). After applying the inclusion and exclusion criteria, 47,776 individuals were excluded due to pregnancy or being under 20 years old, leaving 53,540 participants. Subsequently, 135 participants without follow-up survival data and 46,353 participants without a confirmed asthma diagnosis were excluded, reducing the cohort to 7,052 participants. Of these, 738 participants were excluded for missing LHR data, and 991 were excluded for incomplete covariate information, including key demographic and clinical variables. This stepwise screening process resulted in a final analytic cohort of 5,323 participants. Details of the screening process are presented in Figure 1.

### Definitions and measurements of asthma and LHR

Asthma was identified based on participants’ self-reported responses to the question, “Have you ever been diagnosed with asthma by a doctor?” Participants who answered “Yes” were classified as having asthma.

The LHR was calculated using data collected at NHANES mobile examination centers. Absolute lymphocyte counts were obtained from complete blood count (CBC) analysis performed with a Beckman Coulter automated hematology analyzer and expressed in units of ×10^9^ cells/L. High-density lipoprotein (HDL) cholesterol levels were measured enzymatically using a Roche Modular P chemistry analyzer and reported in mmol/L. LHR was determined by dividing the lymphocyte count by the HDL cholesterol level.

### Determination of mortality outcomes

Mortality data from NHANES were linked to NDI using probabilistic matching, with follow-up through December 31, 2019 (21). The NDI provided information on mortality status and causes of death, classified under the International Classification of Diseases, 10th Revision (ICD-10). All-cause mortality was defined as deaths from any cause. CVD mortality included deaths caused by cardiovascular diseases, identified by ICD-10 codes I00–I09, I11, I13, and I20–I51. CLRD mortality included deaths caused by chronic lower respiratory diseases, identified by ICD-10 codes J40–J47 (22). Follow-up time was defined as the duration from the participant’s initial NHANES examination until death, loss to follow-up, or the study endpoint on December 31, 2019. We used the “MORTSTAT” variable as the status of death and the “PERMTH_EXM” variable as the follow-up time (21). The mean follow-up time was 106.95 ± 63.27 months, based on the complete-case sample.

### Description of covariates

This study included demographic characteristics, lifestyle factors, disease history, and laboratory indicators as covariates. Covariate data were primarily collected through self-reported NHANES interviews and laboratory tests, encompassing gender, age, race, marital status, educational level, family income-to-poverty ratio (PIR), body mass index (BMI), and eosinophils. Race was classified as Non-Hispanic White, Non-Hispanic Black, Mexican American, and Other (including non-Mexican Hispanic and non-Hispanic multiracial populations). Marital status was categorized into two groups: “married or living with partners” and “living alone.” Educational level was grouped based on years of schooling into three categories: below high school (<9 years), high school (9–12 years), and above high school (>12 years). PIR was expressed as the median with interquartile range, serving as a measure of economic status.

Smoking status was divided into three categories based on lifetime cigarette consumption: never smokers (<100 cigarettes), former smokers (≥100 cigarettes but not currently smoking), and current smokers (≥100 cigarettes and currently smoking). Alcohol use was self-reported and categorized as drinkers (defined as having consumed at least 12 alcoholic drinks in their lifetime) or non-drinkers (not meeting this threshold). Hypertension was defined as self-reported diagnosis by a healthcare provider on at least two occasions. Diabetes status was determined by participants’ self-reported responses to whether they had ever been diagnosed with diabetes by a doctor. Atherosclerotic cardiovascular disease (ASCVD) was identified based on participants’ history of coronary heart disease, angina, heart attack, or stroke, consistent with clinical guidelines for ASCVD classification. BMI was calculated as weight (kg) divided by the square of height (m) and was used to evaluate obesity. Eosinophils, expressed as an absolute number per liter of blood (×10^9^/L), was used as a marker of inflammatory status. Prescription drug use was defined as a positive response to the question, “Have you used any prescription drugs in the past month?” Physical activity was assessed using the Global Physical Activity Questionnaire (GPAQ), which covers daily leisure and sedentary activities from 1999 to 2018. The study adopted the NHANES recommended metabolic equivalent (MET) values for various exercises to quantify physical activity. Physical activity levels were calculated based on the MET values, frequency, and duration of activities each week, using the formula: PA (MET-min/week) = MET× weekly frequency× duration of each PA (23).

### Statistical methods

For continuous variables following a normal distribution, data were presented as the mean ± standard deviation (SD), and group differences were assessed using independent-sample t-tests or one-way analysis of variance (ANOVA). Continuous variables with non-normal distributions were described as medians (interquartile ranges, IQRs) and compared using the Wilcoxon rank-sum test. Categorical variables were expressed as frequencies (n) and percentages (%), with group differences analyzed using Pearson’s chi-square (*χ*^2^) test or Fisher’s exact test. Missing data were handled using multiple imputation (10 imputations) via the chained equations method. Predictive mean matching was used for continuous variables, and binary logistic regression was applied for categorical variables (24). The imputation model incorporated covariates, exposure variables, outcome variables, and other relevant variables as predictors. Pooled results from the imputed datasets were used for all analyses.

Cox proportional hazards models were developed to evaluate the associations between LHR and mortality outcomes, including all-cause mortality, CVD mortality, and CLRD mortality. Results were reported as hazard ratios (HRs) with 95% confidence intervals (CIs). The proportional hazards assumption was verified using Schoenfeld residual tests (25), and no violations were observed (Supplementary Table 1). LHR was analyzed both as a continuous variable and as a categorical variable. Three Cox models were constructed: an unadjusted model; Model 1, adjusted for gender, age, race, education level, marital status, PIR, BMI, eosinophils, smoking status and alcohol use; and Model 2, further adjusted for ASCVD, hypertension, diabetes, prescribed medications and PA-MET. Additionally, LHR was categorized into tertiles (T1, T2, T3), and linear trends (P for trend) were evaluated using Cox regression.

Kaplan–Meier survival curves were plotted to compare survival probabilities across LHR tertiles, and differences between groups were assessed using the log-rank test. Furthermore, restricted cubic spline (RCS) models were employed to explore potential nonlinear dose–response relationships between LHR and mortality risk. Knots were placed at the 5th, 50th, and 95th percentiles of the exposure distribution. Analyses were limited to 99% of the data to reduce the influence of outliers. Given that asthma patients may encounter various competing mortality events, the standard Cox model might overestimate the risk of the primary outcome. To overcome this limitation, we employed the Fine-Gray subdistribution hazard model (26), which enables a more accurate estimation of the cumulative incidence by appropriately accounting for competing events.

To assess the consistency of associations between LHR and different mortality outcomes, predefined subgroup analyses were performed. Subgroup variables included gender, age (≤60 vs. >60 years), BMI (<25 vs. ≥25 kg/m^2^), smoking status (never, former, current), alcohol use (drinker vs. non-drinker), ASCVD, hypertension, diabetes, and prescribed medications. Subgroup analyses for all-cause mortality were performed using Cox regression models, while competing risk models were used for CVD mortality and CLRD mortality. Interaction terms were incorporated into subgroup models to test for heterogeneity among subgroups.

To ensure the robustness of the primary findings, multiple sensitivity analyses were conducted. First, multivariate regression analyses were repeated using a complete-case dataset that excluded missing data. Second, analyses were re-run after excluding outliers, defined as LHR values exceeding the mean ± 3 SD. Third, E-values and their corresponding lower confidence intervals were calculated to evaluate the potential impact of unmeasured confounding on the results. The influence of missing data was further assessed by comparing results before and after multiple imputations. These sensitivity analyses confirmed the robustness of the findings under different assumptions and analytic strategies. All statistical analyses were conducted using R software (version 4.2.1; R Foundation for Statistical Computing, https://www.r-project.org) and Free Statistics software (version 2.0; Beijing Free Clinical Medical Technology Co., Ltd) (27). A two-sided *p*-value <0.05 was considered statistically significant.

## Results

### Baseline characteristics

This study included 5,323 participants with a mean age of 47.79 ± 17.64 years, 42.83% of whom were male. The median LHR was 1.59 (interquartile range: 1.15–2.18). Participants were categorized into three groups based on LHR tertiles: T1 (LHR ≤ 1.29, *n* = 1,767), T2 (LHR 1.29–1.94, *n* = 1,772), and T3 (LHR ≥ 1.94, *n* = 1,784). Supplementary Figure 1 illustrates the distribution of LHR across the tertiles, and Table 1 summarizes the baseline characteristics of each tertile group.

**Table 1:** Baseline characteristics stratified by LHR tertiles.

Variable	LHR				*p* value
	Total	T1(0.19–1.29)	T2(1.29–1.94)	T3(1.94–43.50)	
*N*	5,323	1,767	1,772	1,784	
Gender, *n* (%)					< 0.001
Male	2,280 (42.83)	682 (38.6)	779 (43.96)	819 (45.91)	
Female	3,043 (57.17)	1,085 (61.4)	993 (56.04)	965 (54.09)	
Age(years)	47.79 ± 17.64	52.32 ± 18.21	46.23 ± 17.41	44.85 ± 16.36	< 0.001
Race, *n* (%)					< 0.001
Non-Hispanic White	2,656 (49.90)	915 (51.78)	881 (49.72)	860 (48.21)	
Non-Hispanic Black	1,235 (23.20)	433 (24.5)	421 (23.76)	381 (21.36)	
Mexican American	526 (9.88)	143 (8.09)	168 (9.48)	215 (12.05)	
Other	906 (17.02)	276 (15.62)	302 (17.04)	328 (18.39)	
Education level, *n* (%)					< 0.001
Below high school	426 (8.00)	135 (7.64)	125 (7.05)	166 (9.3)	
High school	1,937 (36.39)	565 (31.98)	665 (37.53)	707 (39.63)	
Above high school	2,960 (55.61)	1,067 (60.38)	982 (55.42)	911 (51.07)	
Marital Status, *n* (%)					0.174
Married or living with partners	2,898 (54.44)	942 (53.31)	953 (53.78)	1,003 (56.22)	
Living alone	2,425 (45.56)	825 (46.69)	819 (46.22)	781 (43.78)	
Smoking status, *n* (%)					< 0.001
Current	1,300 (24.42)	304 (17.2)	375 (21.16)	621 (34.81)	
Former	1,424 (26.75)	509 (28.81)	518 (29.23)	397 (22.25)	
Never	2,599 (48.83)	954 (53.99)	879 (49.6)	766 (42.94)	
Alcohol use, *n* (%)					0.025
No	1,326 (24.91)	428 (24.22)	414 (23.36)	484 (27.13)	
Yes	3,997 (75.09)	1,339 (75.78)	1,358 (76.64)	1,300 (72.87)	
Hypertension, *n* (%)					0.407
No	3,460 (65.00)	1,163 (65.82)	1,159 (65.41)	1,138 (63.79)	
Yes	1863 (35.00)	604 (34.18)	613 (34.59)	646 (36.21)	
Diabetes, *n* (%)					< 0.001
No	4,516 (84.84)	1,554 (87.95)	1,525 (86.06)	1,437 (80.55)	
Yes	807 (15.16)	213 (12.05)	247 (13.94)	347 (19.45)	
Prescribed medications, *n* (%)					< 0.001
No	1,572 (29.53)	450 (25.47)	565 (31.88)	557 (31.22)	
Yes	3,751 (70.47)	1,317 (74.53)	1,207 (68.12)	1,227 (68.78)	
ASCVD, *n* (%)					0.013
No	4,588 (86.19)	1,526 (86.36)	1,556 (87.81)	1,506 (84.42)	
Yes	735 (13.81)	241 (13.64)	216 (12.19)	278 (15.58)	
PA-MET	600.00 (0.00,2640.00)	571.67 (0.00,2400.00)	720.00 (0.00,2817.85)	540.00 (0.00,2880.00)	0.155
PIR	1.94 (1.02, 3.94)	2.29 (1.18, 4.54)	2.04 (1.03, 3.87)	1.60 (0.89, 3.40)	< 0.001
BMI, (kg/m^2^)	30.52 ± 8.03	27.99 ± 7.06	30.66 ± 7.90	32.87 ± 8.34	< 0.001
Eosinophils, ×10^9^/L	0.24 ± 0.18	0.21 ± 0.16	0.24 ± 0.19	0.27 ± 0.20	< 0.001
LHR	1.59 (1.15, 2.18)	1.00 (0.82, 1.15)	1.59 (1.44, 1.75)	2.48 (2.18, 3.06)	< 0.001
All-cause Mortality					< 0.001
Alive	4,599 (86.40)	1,456 (82.4)	1,561 (88.09)	1,582 (88.68)	
Death	724 (13.60)	311 (17.6)	211 (11.91)	202 (11.32)	
CVD mortality					0.004
Alive	5,140 (96.56)	1,687 (95.47)	1714 (96.73)	1739 (97.48)	
Death	183 (3.44)	80 (4.53)	58 (3.27)	45 (2.52)	
CLRD mortality					< 0.001
Alive	5,232 (98.29)	1720 (97.34)	1753 (98.93)	1759 (98.6)	
Death	91 (1.71)	47 (2.66)	19 (1.07)	25 (1.4)	
Follow up time	106.95 ± 63.27	103.26 ± 63.95	109.94 ± 63.77	107.63 ± 61.94	0.006

Data are presented as mean (SD) for continuous variables and percent for categorical variables. This table is based on the complete-case sample (*n* = 5,323), while subsequent analyses used multiple imputation with a total sample size of 6,314. Missing data ranged from 0.05 to 7.68% across variables. Notably, PIR had the highest missing rate (7.68%). Other variables (e.g., education, marital status, BMI, smoking, etc.) had minimal missing rates (<2%). Multiple imputation was applied to address missing data in the analyses. LHR, lymphocyte-to-high-density lipoprotein cholesterol ratio; PIR, Ratio of family income to poverty; ASCVD, Atherosclerotic Cardiovascular Disease; BMI, body mass index; PA-MET physical activity metabolic equivalent; CVD, Cardiovascular Disease; CLRD, Chronic Lower Respiratory Disease.

Significant differences (*p* < 0.05) were observed among the tertile groups in variables such as gender, age, race, educational level, smoking status, alcohol use, diabetes, prescription medications, ASCVD, PIR, BMI, and eosinophils. Participants in the higher LHR tertiles were younger, had higher BMI, a greater proportion of current smokers, higher prevalence of diabetes and ASCVD, elevated eosinophil counts, and lower PIR values.

During a mean follow-up period of 106.95 ± 63.27 months (maximum: 228 months), 724 all-cause deaths (13.60%) were recorded, including 183 deaths from CVD (3.44%) and 91 deaths from CLRD (1.71%). The baseline characteristics of participants stratified by all-cause, CVD, and CLRD mortality are detailed in Supplementary Tables 2–4.

### Associations between LHR and mortality

Table 2 presents the results of the Cox proportional hazards regression analysis examining the association between LHR and mortality in patients with asthma. In the fully adjusted model (Model 2), after accounting for potential confounders, LHR levels were significantly inversely associated with all-cause mortality, CVD mortality, and CLRD mortality. Specifically, each one-unit increase in log₂ LHR was associated with a 18% reduction in the risk of all-cause mortality (HR = 0.82, 95% CI: 0.74–0.91, *p* < 0.001), a 21% reduction in the risk of CVD mortality (HR = 0.79, 95% CI: 0.65–0.96, *p* = 0.018), and a 41% reduction in the risk of CLRD mortality (HR = 0.59, 95% CI: 0.45–0.77, *p* < 0.001). Subgroup analyses showed that, compared to the lowest tertile (T1), participants in the highest tertile (T3) experienced a 23% reduction in the risk of all-cause mortality (HR = 0.77, 95% CI: 0.65–0.92, *p* = 0.003) and a 37% reduction in the risk of CVD mortality (HR = 0.63, 95% CI: 0.44–0.91, *p* = 0.014). For CLRD mortality, participants in the middle tertile (T2) exhibited a 55% lower risk compared to the lowest tertile (T1) (HR = 0.45, 95% CI: 0.27–0.73, *p* = 0.002), whereas no significant association was observed for the highest tertile (T3) (HR = 0.66, 95% CI: 0.41–1.08, *p* = 0.100). Trend tests revealed statistically significant results (P for trend <0.05).

**Table 2:** Multivariate analysis of the association between LHR and mortality outcomes in asthma patients.

LHR	Events (%)	Crude Model		Model 1		Model 2	
		HR (95% CI)	*p*-value	HR (95% CI)	*p*-value	HR (95% CI)	*p*-value
All-cause mortality							
Log_2_(LHR)	905 (14.3)	0.68 (0.62 ~ 0.75)	<0.001	0.85 (0.77–0.94)	0.001	0.82 (0.74–0.91)	<0.001
LHR category							
T1	390 (18.6)	1(Ref)		1(Ref)		1(Ref)	
T2	270 (12.8)	0.65 (0.55 ~ 0.75)	<0.001	0.86 (0.73 ~ 1.01)	0.057	0.83 (0.71 ~ 0.98)	0.027
T3	245 (11.6)	0.59 (0.50 ~ 0.69)	<0.001	0.82 (0.69 ~ 0.98)	0.025	0.77 (0.65 ~ 0.92)	0.003
*P* for trend			<0.001		0.020		0.003
CVD mortality							
Log_2_(LHR)	229 (3.6)	0.67 (0.56 ~ 0.81)	<0.001	0.83 (0.68 ~ 1.01)	0.062	0.79 (0.65 ~ 0.96)	0.018
LHR category							
T1	99 (4.7)	1(Ref)		1(Ref)		1(Ref)	
T2	76 (3.6)	0.72 (0.53 ~ 0.97)	0.032	0.99 (0.72 ~ 1.35)	0.932	0.94 (0.69 ~ 1.28)	0.693
T3	54 (2.6)	0.51 (0.37 ~ 0.72)	<0.001	0.70 (0.49 ~ 1.00)	0.054	0.63 (0.44 ~ 0.91)	0.014
*P* for trend			<0.001		0.065		0.017
CLRD mortality							
Log_2_(LHR)	115 (1.8)	0.46 (0.35 ~ 0.60)	<0.001	0.59 (0.45 ~ 0.77)	<0.001	0.59 (0.45 ~ 0.77)	<0.001
LHR category							
T1	62 (3.0)	1(Ref)		1(Ref)		1(Ref)	
T2	23 (1.1)	0.34 (0.21 ~ 0.56)	<0.001	0.45 (0.28 ~ 0.74)	0.002	0.45 (0.27 ~ 0.73)	0.002
T3	30 (1.4)	0.45 (0.29 ~ 0.70)	0.001	0.68 (0.42 ~ 1.09)	0.111	0.66 (0.41 ~ 1.08)	0.100
*P* for trend			<0.001		0.047		0.040

Crude Model, unadjusted; Model 1, Adjusted for gender, age, race, education level, marital status, PIR, BMI, Eosinophils, alcohol use, smoking status; Model 2, Further adjusted for ASCVD, hypertension, diabetes, Prescribed medications, PA-MET; LHR, lymphocyte-to-high-density lipoprotein cholesterol ratio; CVD, Cardiovascular Disease; PIR, Ratio of family income to poverty; ASCVD, Atherosclerotic Cardiovascular Disease; BMI, body mass index; PA-MET, physical activity metabolic equivalent; Ref:Reference; CLRD, Chronic Lower Respiratory Disease.

Kaplan–Meier survival curves illustrated significant differences in survival probabilities among the LHR tertile groups (T1, T2, T3) (Supplementary Figure 2). The log-rank test further confirmed that these group differences were statistically significant (*p* < 0.05).

### Dose–response relationship between LHR and mortality

**Figure 2:**
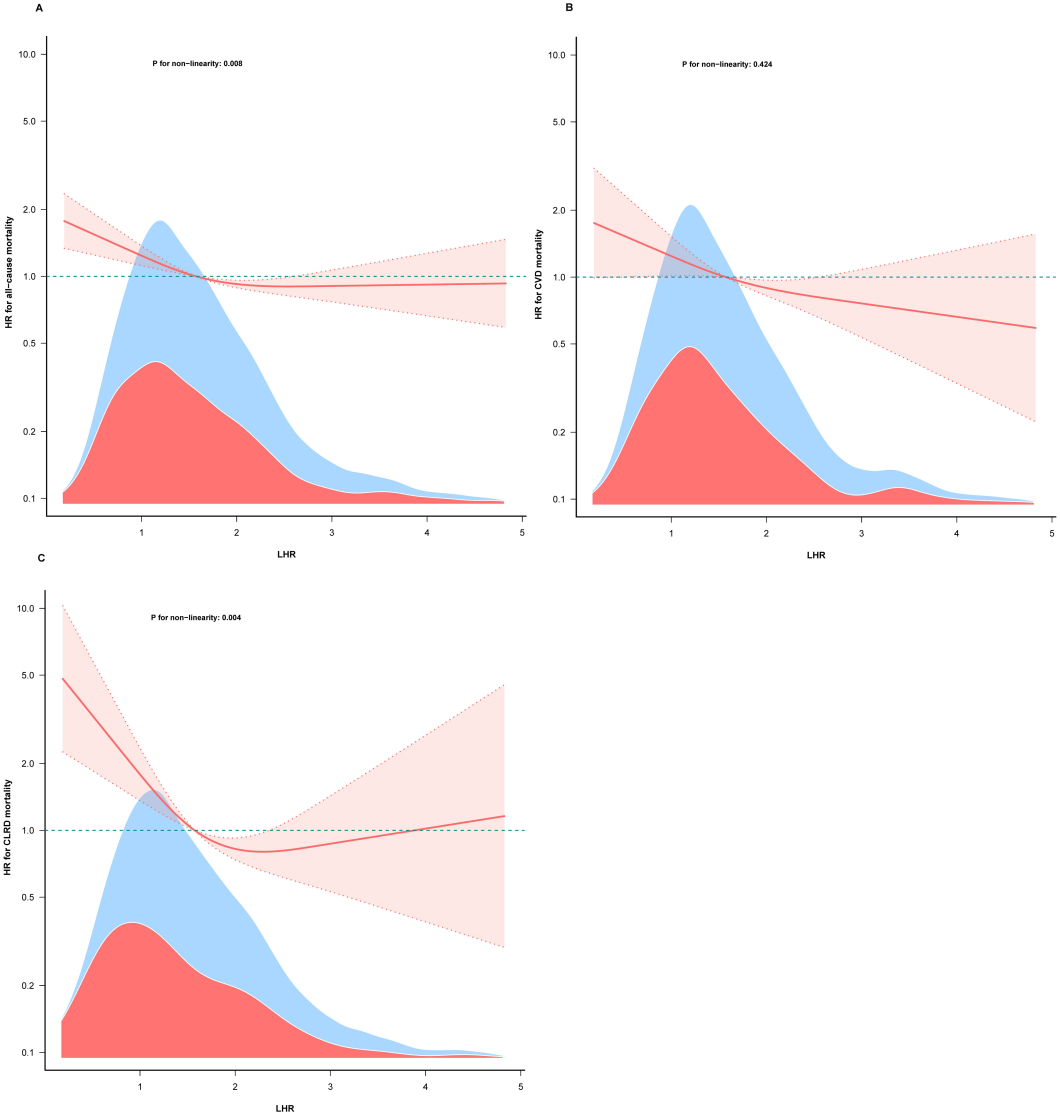
The association of LHR with all-cause **(A)**, CVD **(B)**, and CLRD mortality **(C)** among asthma patients visualized by restricted cubic spline. Hazard ratios were adjusted for gender, age, race, education level, marital status, PIR, BMI, Eosinophils, alcohol use, smoking status, ASCVD, hypertension, diabetes, Prescribed medications, PA-MET.

RCS analyses were conducted to assess the dose–response relationship between LHR and all-cause mortality, and CVD mortality, CLRD mortality (Figures 2A–C). The results revealed that LHR was associated with a characteristic L-shaped curve for all-cause mortality and CLRD mortality, while its relationship with CVD mortality exhibited a linear trend. Non-linearity tests confirmed statistically significant non-linear associations for all-cause mortality and CLRD mortality (non-linearity *p* < 0.05), whereas no significant non-linearity was observed for CVD mortality (non-linearity *p* = 0.424).

Threshold effect analyses further identified inflection points at LHR = 1.78 for all-cause mortality and LHR = 1.52 for CLRD mortality. Below these inflection points, each 1-unit increase in LHR was associated with a 40% reduction in the risk of all-cause mortality (HR = 0.60, 95% CI: 0.47–0.77, *p* < 0.001) and an 85% reduction in the risk of CLRD mortality (HR = 0.15, 95% CI: 0.07–0.33, p < 0.001). However, above the inflection points, no significant associations were observed (all-cause mortality: HR = 0.96, 95% CI: 0.84–1.10, *p* = 0.565; CLRD mortality: HR = 1.06, 95% CI: 0.80–1.42, *p* = 0.675). The log-likelihood ratio test further validated the non-linear relationships for all-cause mortality and CLRD mortality (*p* < 0.05, Table 3). In contrast, the dose–response relationship between LHR and CVD mortality followed a linear pattern. As demonstrated by the RCS analysis (non-linearity *p* = 0.424, Figure 2B), the multivariable Cox regression analysis indicated that each 1-unit increase in log₂ LHR was associated with a 21% reduction in the risk of CVD mortality (HR = 0.79, 95% CI: 0.65–0.96, *p* = 0.018).

**Table 3:** Threshold effect of LHR on all-cause and CLRD mortality.

LHR	Adjusted HR (95% CI)	*p*-value
With all-cause mortality		
LHR < 1.78	0.60 (0.47–0.77)	<0.001
LHR ≥ 1.78	0.96 (0.84–1.10)	0.565
Log-likelihood ratio test		0.001
CLRD mortality		
LHR < 1.52	0.15 (0.07–0.33)	<0.001
LHR ≥ 1.52	1.06 (0.80–1.42)	0.675
Log-likelihood ratio test		<0.001

Adjusted for gender, age, race, education level, marital status, PIR, BMI, Eosinophils, alcohol use, smoking status, ASCVD, hypertension, diabetes, Prescribed medications; PA-MET; LHR, lymphocyte-to-high-density lipoprotein cholesterol ratio; PIR, Ratio of family income to poverty; ASCVD, Atherosclerotic Cardiovascular Disease; BMI, body mass index; PA-MET, physical activity metabolic equivalent; CLRD, Chronic Lower Respiratory Disease.

### Competing risk analysis via the fine-gray model

**Figure 3:**
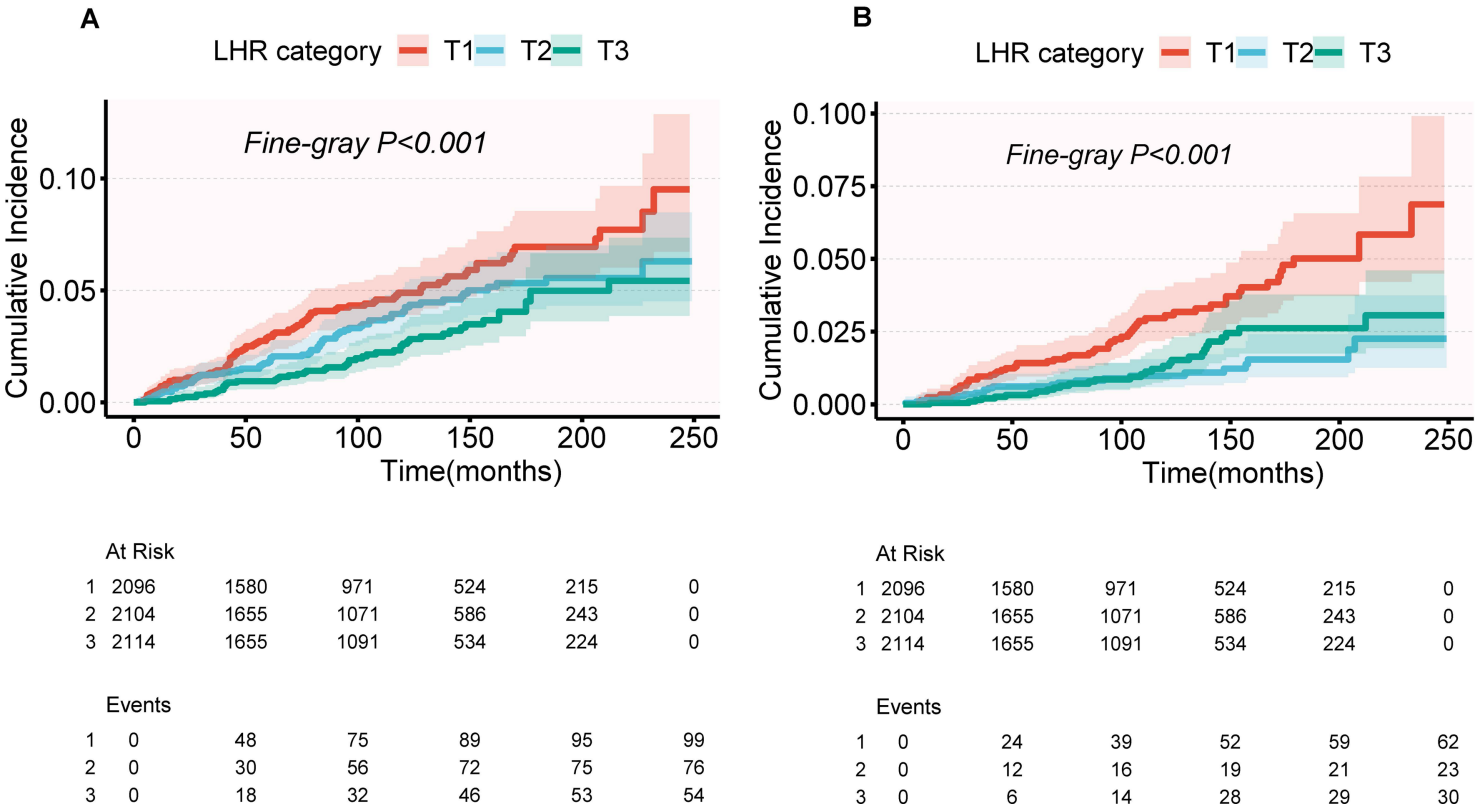
Cumulative incidence of CVD mortality **(A)** and CLRD mortality **(B)** across LHR categories (T1, T2, T3) using a competing risk model. Cumulative incidence functions were estimated using Fine-Gray subdistribution hazard models. *p*-values for Fine-Gray tests: *p* < 0.001 for both CVD mortality **(A)** and CLRD mortality **(B)**.

As shown in Figure 3, the Fine-Gray competing risk model revealed that individuals in the highest LHR tertile (T3) had significantly lower cumulative risks of CVD mortality and CLRD mortality compared to those in the lowest tertile (T1) over the follow-up period (*p* < 0.001, Figure 3).

For CVD mortality, after multivariable adjustment (Model 2), the protective association in the T3 group did not reach statistical significance (SHR = 0.70, 95% CI: 0.49–1.00, *p* = 0.051), and the trend test was also non-significant (P for trend = 0.062) (Table 4). The cumulative risk curves indicated that the T3 group had a lower cumulative risk of CVD mortality than the T1 group in the later stages of follow-up (>200 months), with the difference widening over time (Figure 3A). For CLRD mortality, after multivariable adjustment (Model 2), LHR log₂ as a continuous variable was significantly associated with a lower risk of CLRD mortality (SHR = 0.64, 95% CI: 0.46–0.88, *p* = 0.007). In the categorical analysis, the T2 group showed a significantly reduced risk of CLRD mortality compared to the T1 group (SHR = 0.50, 95% CI: 0.30–0.83, *p* = 0.007), whereas no significant reduction was observed in the T3 group (SHR = 0.73, 95% CI: 0.43–1.22, *p* = 0.232). The trend test for CLRD mortality was non-significant (P for trend = 0.146). The cumulative risk curves showed that the T3 group had the most pronounced reduction in CLRD mortality risk during the mid-follow-up period (100–200 months) compared to the T1 group (Table 4, Figure 3).

**Table 4:** Association of LHR with CVD and CLRD mortality using fine-gray model.

LHR	Events (%)	Crude Model		Model 1		Model 2	
		SHR (95%CI)	*p*-value	SHR (95%CI)		SHR (95%CI)	*p*-value
CVD mortality							
Log_2_(LHR)	229 (3.6)	0.70 (0.58 ~ 0.86)	0.001	0.86 (0.71 ~ 1.04)	0.114	0.85 (0.70 ~ 1.03)	0.093
LHR category							
T1	99 (4.7)	1(Ref)		1(Ref)		1(Ref)	
T2	76 (3.6)	0.75 (0.56 ~ 1.01)	0.060	1.04 (0.76 ~ 1.42)	0.816	1.01 (0.74 ~ 1.38)	0.969
T3	54 (2.6)	0.54 (0.38 ~ 0.75)	<0.001	0.72 (0.51 ~ 1.02)	0.062	0.70 (0.49 ~ 1.00)	0.051
*P* for trend			<0.001		0.076		0.062
CLRD mortality							
Log_2_(LHR)	115 (1.8)	0.49 (0.36 ~ 0.68)	<0.001	0.63 (0.46 ~ 0.87)	0.005	0.64 (0.46 ~ 0.88)	0.007
LHR category							
T1	62 (3.0)	1(Ref)		1(Ref)		1(Ref)	
T2	23 (1.1)	0.36 (0.22 ~ 0.58)	<0.001	0.50 (0.30 ~ 0.82)	0.007	0.50 (0.30 ~ 0.83)	0.007
T3	30 (1.4)	0.48 (0.31 ~ 0.74)	0.001	0.71 (0.43 ~ 1.19)	0.195	0.73 (0.43 ~ 1.22)	0.232
*P* for trend			0.001		0.122		0.146

Crude Model, unadjusted; Model 1, Adjusted for gender, age, race, education level, marital status, PIR, BMI, Eosinophils, alcohol use, smoking status; Model 2, Further adjusted for ASCVD, hypertension, diabetes, Prescribed medications, PA-MET; LHR, lymphocyte-to-high-density lipoprotein cholesterol ratio; CVD, Cardiovascular Disease; PIR, Ratio of family income to poverty; ASCVD, Atherosclerotic Cardiovascular Disease; BMI, body mass index; PA-MET, physical activity metabolic equivalent; Ref:Reference; CLRD, Chronic Lower Respiratory Disease.

### Subgroup analysis

**Figure 4:**
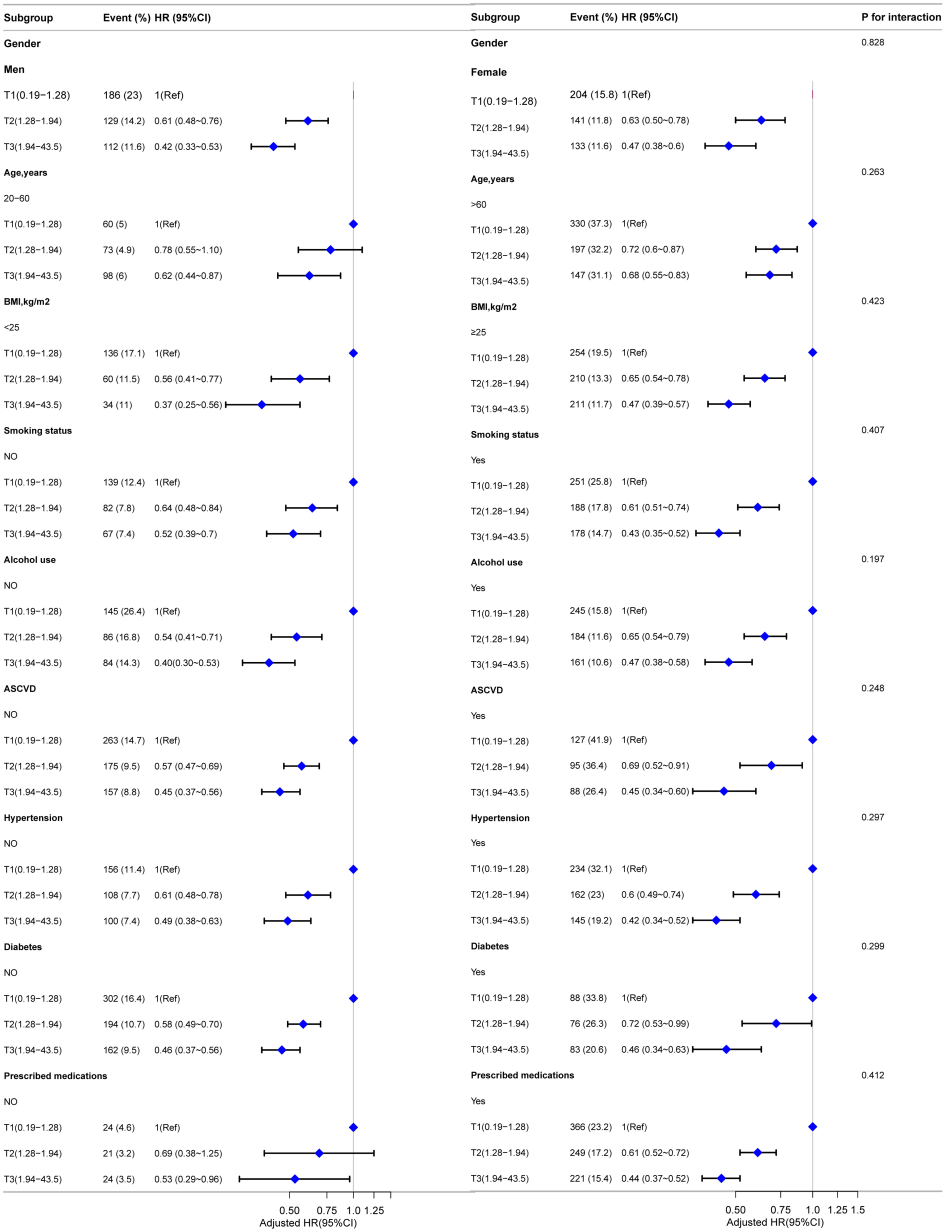
Forest plot for subgroup analysis of association between LHR and all-cause mortality. Except for the stratification factor itself, the stratified analysis was adjusted for all variables. Multivariate Cox regression models were used to calculate hazard ratios (HRs), and interaction *p*-values were provided for each subgroup.

**Figure 5:**
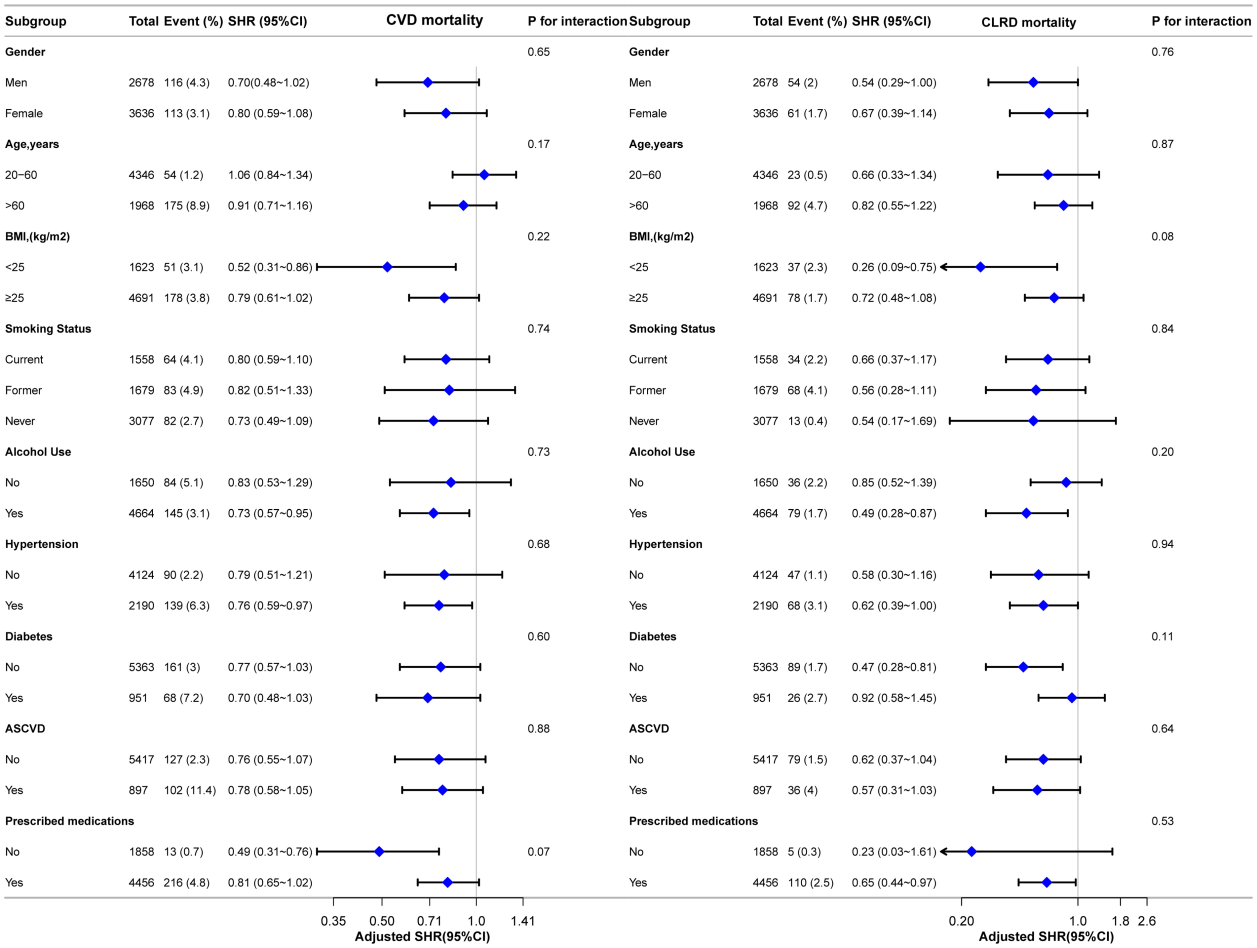
Forest plot for subgroup analysis of the association between LHR and CVD and CLRD mortality using competing risk models. Subdistribution hazard ratios (HRs) were calculated using Fine-Gray competing risk models. Except for the stratification factor itself, the stratified analysis was adjusted for all variables. Interaction p-values were provided for each subgroup.

Subgroup analyses were conducted to explore the associations between LHR and the risks of all-cause mortality, CVD mortality, and CLRD mortality. These analyses were stratified by gender, age, BMI, smoking status, alcohol use, ASCVD, hypertension, diabetes status, and prescribed medications. Traditional Cox regression models were employed to assess all-cause mortality, while competing risk models were applied for CVD mortality and CLRD mortality. The results demonstrated consistent associations between LHR and all mortality outcomes across all subgroups. Furthermore, no significant effect modifications were observed in the interaction analyses (all interaction *p*-values >0.05) (Figures 4, 5).

### Sensitivity analysis

To ensure the robustness of the findings, several sensitivity analyses were performed. First, multivariate regression analyses were conducted on a complete-case dataset (*n* = 5,323) that excluded all missing data. These analyses confirmed the associations between LHR and the risks of all-cause mortality, CVD mortality, and CLRD mortality, consistent with the primary findings (Supplementary Table 5). Second, after excluding outliers with LHR values exceeding the mean ± 3 standard deviations (*n* = 6,274), the significant associations between LHR and the specified mortality outcomes were further corroborated (Supplementary Table 6). Finally, E-value calculations were conducted to evaluate the potential influence of unmeasured confounders. The results indicated that the observed associations between LHR and mortality risks remained robust even in the presence of hypothetical unmeasured confounding (Supplementary Table 7).

In addition, Supplementary Figure 3 demonstrates the consistent associations between Log_2_(LHR) and the risks of all-cause mortality, CVD mortality, and CLRD mortality across 10 imputed datasets. These findings mitigate concerns about the impact of missing data. Supplementary Figure 4 illustrates the distribution of missing values among study variables, which showed a relatively low proportion of missing data without evidence of significant bias. These results validate the appropriateness of the multiple imputation method and further support the robustness of the study conclusions.

## Discussion

This study, based on population data from NHANES (1999–2018), investigated the association between the LHR and mortality risk in asthma patients. After multivariable adjustment, higher LHR levels were significantly associated with reduced risks of all-cause mortality, CVD mortality, and CLRD mortality in asthma patients. Dose–response analyses revealed an L-shaped nonlinear relationship between LHR and both all-cause mortality and CLRD mortality, with more pronounced protective effects at lower LHR levels, while a linear relationship was observed for CVD mortality. The robustness of these associations, particularly the protective effect of LHR against CLRD mortality, was confirmed using competing risk models. Subgroup analyses further demonstrated that this protective association remained consistent across various population subgroups. This study highlights the potential value of LHR as an inflammation-immune biomarker in mortality risk assessment among asthma patients and provides theoretical support for its clinical application.

Previous studies support the primary findings of this research. As a biomarker reflecting both lipid metabolism and inflammatory status, LHR has demonstrated significant prognostic value in various chronic diseases (28). Liu et al. (15) identified LHR ≤ 0.6 as a significant predictor of mortality in sepsis patients, with a 90-day mortality rate markedly higher in patients with low LHR (OR = 1.74, *p* = 0.001). Moreover, lower LHR levels were associated with a nonlinear increase in mortality risk (*p* < 0.001). In COPD patients, LHR has been shown to correlate positively with lung function (FEV1/FVC ratio, *r* = 0.42, *p* < 0.001) and is significantly associated with an increased risk of all-cause mortality at lower levels (19). Similarly, LHR has demonstrated robust prognostic utility in patients with diabetes and metabolic syndrome (11, 28). In asthma, current mortality risk assessments primarily rely on markers of localized airway inflammation. For example, Price et al. (29), in a large-scale study (*n* = 12,563), found that elevated peripheral blood eosinophil counts (>400 cells/μL) were significantly associated with increased mortality risk (HR = 1.42, *p* < 0.05). While the neutrophil-to-lymphocyte ratio (NLR) has shown some predictive value (30), these traditional markers are limited to assessing localized inflammation. In contrast, this study is the first to reveal that LHR not only predicts all-cause mortality in asthma patients but is also specifically associated with mortality risks from chronic lower respiratory disease and cardiovascular disease. These findings underscore the unique value of LHR in evaluating systemic inflammation and metabolic status, offering a more comprehensive approach to risk assessment in asthma patients.

This study found that the LHR is significantly associated with the mortality risk in asthma patients, and its potential mechanisms may involve multiple molecular pathways. As a comprehensive indicator, LHR reflects the real-time dynamic changes in the body’s immune status and inflammatory response (15, 31). In asthma immune regulation, lymphocytes include not only T cells and B cells, which play adaptive immune roles, but also natural killer cells and other innate lymphoid cells with innate immune functions (31, 32). Although the traditional view attributes asthma to Th2 cell-mediated inflammation—where Th2 cells induce eosinophil infiltration and IgE production by secreting IL-4, IL-5, and IL-13 (33, 34)—studies have shown that some patients may exhibit non-T2-type inflammation, such as immune responses mediated by Th1 and Th17 cells [31a]. Furthermore, regulatory T cells (Tregs) also play a crucial role in suppressing inflammation and maintaining immune homeostasis (35). Therefore, low lymphocyte counts not only indicate weakened adaptive immune function but may also reflect insufficient innate immune regulatory capacity, thereby exacerbating airway inflammation, causing tissue damage, accelerating disease progression, and increasing the risk of death (36–38).

HDL, known for its anti-inflammatory and antioxidant properties, has demonstrated protective effects in asthma (39). HDL mitigates airway inflammation and oxidative stress via multiple mechanisms, including the clearance of inflammatory mediators, inhibition of inflammatory cell activation, reduction of pro-inflammatory factor release, and maintenance of airway epithelial barrier integrity (40, 41). However, reduced HDL levels and functional impairment, common in asthma patients, may aggravate systemic inflammation (42). This study’s finding that lower LHR levels are significantly associated with increased mortality risk suggests a synergistic effect of lymphocyte reduction and HDL dysfunction in driving systemic inflammatory imbalance, ultimately leading to higher mortality. Although physical exercise can enhance HDL levels and lower mortality risk (43), our analysis adjusting for PA-MET revealed that the association between LHR and mortality remained robust, indicating that the results are not driven solely by differences in physical activity.

Further analysis revealed that the relationship between LHR and specific mortality types involves distinct mechanisms. For CLRD mortality, low LHR levels reflect immune-inflammatory imbalance, which may promote airway remodeling, pulmonary fibrosis, and lung function decline, increasing the risk of death (44). For CVD mortality, HDL dysfunction is a key factor, contributing to atherosclerosis progression, endothelial dysfunction, and inflammation-mediated thrombosis (45). Additionally, reduced LHR levels may heighten all-cause mortality risk by impairing immune defense, intensifying oxidative stress, and disrupting tissue repair (15).

By integrating the biological effects of lymphocytes and HDL, LHR reflects the dynamic changes in the inflammatory and immune status in asthma patients, revealing its potential biological mechanisms associated with mortality risk (46, 47). This finding suggests that, when combined with traditional clinical indicators, LHR could support the precise identification of high-risk patients, while facilitating the development of personalized treatment plans through dynamic monitoring of inflammation and immune status, and playing a role in efficacy assessment and prognosis prediction. Although these potential applications require further validation through large-scale studies, this research lays the foundation for exploring the clinical value of LHR in asthma management.

LHR shows promise as a practical and accessible biomarker for assessing asthma-related mortality risk, as it is derived from routine complete blood counts. Unlike conventional biomarkers such as blood eosinophils or FeNO—which primarily reflect localized airway inflammation—LHR captures systemic inflammation and immune-metabolic balance, offering a more comprehensive risk assessment (5, 6). Notably, LHR’s significant associations with both CLRD and CVD mortality distinguish it from traditional markers with a narrower focus. This broader predictive utility could enable clinicians to identify high-risk patients earlier and implement targeted interventions, including closer monitoring and better management of comorbid conditions such as cardiovascular disease (7). Future research should focus on standardizing LHR thresholds and conducting comparative studies with existing biomarkers to establish its clinical accuracy, prognostic value, and utility in routine asthma management.

This study has several strengths. First, the use of the Fine-Gray competing risk model to evaluate the association between LHR and cause-specific mortality provides a more accurate risk assessment by accounting for multiple competing threats. By mitigating the overestimation inherent in traditional survival models, this approach bolsters the robustness of our findings, even though it relies on certain model assumptions and remains sensitive to unmeasured confounders, warranting further validation in future research (26). Second, the application of restricted cubic spline analysis revealed the nonlinear relationship between LHR and mortality risk and quantified the inflection points for all-cause mortality and CLRD mortality (1.78 and 1.52, respectively). Third, the robustness of the results was confirmed through systematic subgroup analyses and multiple sensitivity analyses.

However, this study also has several limitations. First, as an observational study based on the NHANES database, it is difficult to establish a causal relationship between LHR and mortality risk, even after adjusting for known confounders. Second, as a single physiological marker, LHR is susceptible to fluctuations caused by acute infections, therapeutic interventions, or other pathological conditions, which may affect its predictive value. Third, this study relied on self-reported physician-diagnosed asthma, which may introduce recall bias or misclassification. Furthermore, the NHANES database lacks critical clinical information, such as asthma severity, specific treatment regimens, and medication adherence, preventing a more detailed investigation of how these factors might modulate the association between LHR and mortality risk. In addition, treatments like inhaled corticosteroids or bronchodilators may alter LHR levels through their anti-inflammatory effects, while differing asthma severity might independently impact both biomarker levels and outcomes, thereby introducing residual confounding. Moreover, the heterogeneity of asthma phenotypes and the variability in treatment strategies among participants further limit the stratification and adjustment for these factors. Fourth, this study did not track dynamic changes in LHR or classify asthma phenotypes, which limits the evaluation of its utility in specific patient subgroups.

In future research, prospective cohort studies incorporating comprehensive clinical data are essential to further validate the association between LHR and mortality risk. Furthermore, well-designed interventional studies are warranted to determine whether deliberate modulation of LHR levels can directly improve clinical outcomes, thereby reinforcing its utility as a prognostic biomarker. These research approaches would address current limitations regarding the heterogeneity of asthma phenotypes and treatment variations, ultimately paving the way for more personalized and effective management strategies.

## Conclusion

After adjusting for multiple variables, we found a significant nonlinear relationship between LHR and mortality risks in asthma patients. Both all-cause mortality and CLRD mortality showed L-shaped associations with inflection points at 1.78 and 1.52, respectively. Below these thresholds, lower LHR levels were associated with significantly increased mortality risks. Competing risk analysis further validated the significant association between LHR and CLRD mortality risk (SHR = 0.64, 95% CI: 0.46–0.88). These findings suggest that LHR may serve as a potential biomarker for mortality risk assessment in asthma patients, though prospective studies are needed for further validation.

## Data Availability

Publicly available datasets were analyzed in this study. This data can be found at: https://www.cdc.gov/nchs/nhanes/index.htm.
