# Supplementary material for: Lymphocyte-to-high-density lipoprotein ratio and mortality in asthma patients: a novel immunoinflammatory biomarker with nonlinear association
Source: Front Med (Lausanne). 2025 Jun 13;12:1553188. doi: 10.3389/fmed.2025.1553188 (PMC12202334; doi:10.3389/fmed.2025.1553188)
Supplement: Supplementary file 1 [file Table_1.docx]

Supplementary Material

**1. Supplementary Table 1**. Results of the Proportional Hazards Assumption Testing for Cox Models

**2. Supplementary Figure 1** Density Plot of LHR Tertile Groups

**3. Supplementary Table 2.** Baseline characteristics according to all-cause mortality

**4. Supplementary Table 3.** Baseline characteristics according to CVD mortality

**5. Supplementary Table 4.** Baseline characteristics according to CLRD mortality

**6. Supplementary Figure 2** Kaplan–Meier Curves for All-Cause (**A**), CVD(**B**), and CLRD Mortality (**C)** by LHR Tertiles

**7. Supplementary Table 5.** Multivariate analysis of the association between LHR and all-cause, CVD, and CLRD mortality in asthma after complete-case analysis(**N=5323**)

**8. Supplementary Table 6.** Multivariate analysis of the association between LHR and all-cause, CVD, and CLRD mortality in asthma after removing outliers beyond 3 standard deviations (**N=6274**)

**9. Supplementary Table 7.** Association between LHR and all-cause, CVD, and CLRD mortality with E-values for robustness evaluation(**N=6314**)

**10. Supplementary Figure 3**. Results for Multiple Imputations (10 Times) for All-Cause Mortality, CVD Mortality, and CLRD Mortality

**11. Supplementary Figure 4** Distribution and Proportions of Missing Values Across Variables

**Supplementary Table 1**. Results of the Proportional Hazards Assumption Testing for Cox Models

| **Mortality** | **All-cause Mortality** | | **CVD Mortality** | | **CLRD Mortality** | |
| --- | --- | --- | --- | --- | --- | --- |
| **Variable** | Chi-square(χ²) | **p-value**  5.77-6.12 | Chi-square(χ²) | **p-value**  >6.48 | Chi-square(χ²) | **p-value** |
| Gender | 4.208 | 0.040 | 3.507 | 0.061 | 0.051 | 0.822 |
| Age | 1.368 | 0.242 | 0.462 | 0.497 | 0.356 | 0.551 |
| Race | 2.790 | 0.425 | 9.768 | 0.021 | 3.157 | 0.368 |
| Education level | 1.845 | 0.398 | 1.727 | 0.422 | 0.351 | 0.839 |
| Marital Status | 1.060 | 0.303 | 0.001 | 0.971 | 0.361 | 0.548 |
| Smoking status | 3.466 | 0.177 | 0.866 | 0.649 | 1.345 | 0.246 |
| Alcohol use | 0.096 | 0.757 | 2.045 | 0.153 | 0.566 | 0.452 |
| Hypertension | 0.999 | 0.318 | 0.002 | 0.968 | 3.922 | 0.048 |
| Diabetes | 1.668 | 0.197 | 0.210 | 0.647 | 0.089 | 0.765 |
| Prescribed medications | 0.039 | 0.844 | 0.360 | 0.549 | 0.852 | 0.356 |
| ASCVD | 0.175 | 0.676 | 0.574 | 0.449 | 2.887 | 0.089 |
| PIR | 0.043 | 0.835 | 1.261 | 0.261 | 0.783 | 0.376 |
| BMI | 0.004 | 0.948 | 2.165 | 0.141 | 4.884 | 0.027 |
| Eosinophils | 0.041 | 0.840 | 0.258 | 0.611 | 0.756 | 0.385 |
| PA-MET | 0.101 | 0.750 | 1.733 | 0.188 | 8.792 | 0.003 |
| LHR | 0.236 | 0.627 | 2.850 | 0.091 | 2.096 | 0.351 |
| **GLOBAL** | 27.789 | **0.114** | 27.661 | **0.118** | 30.859 | **0.057** |

**Note:** The proportional hazards assumption was assessed using Schoenfeld residual tests. This table presents the results for all-cause mortality, cardiovascular disease (CVD) mortality, and chronic lower respiratory disease (CLRD) mortality models. GLOBAL represents the overall test for all variables combined. p-values greater than 0.05 indicate that the proportional hazards assumption was not violated.

**Abbreviations:** LHR, lymphocyte-to-high-density lipoprotein cholesterol ratio; PIR, Ratio of family income to poverty; ASCVD, Atherosclerotic Cardiovascular Disease; BMI, body mass index; PA-MET physical activity metabolic equivalent; CVD, Cardiovascular Disease; CLRD, Chronic Lower Respiratory Disease;


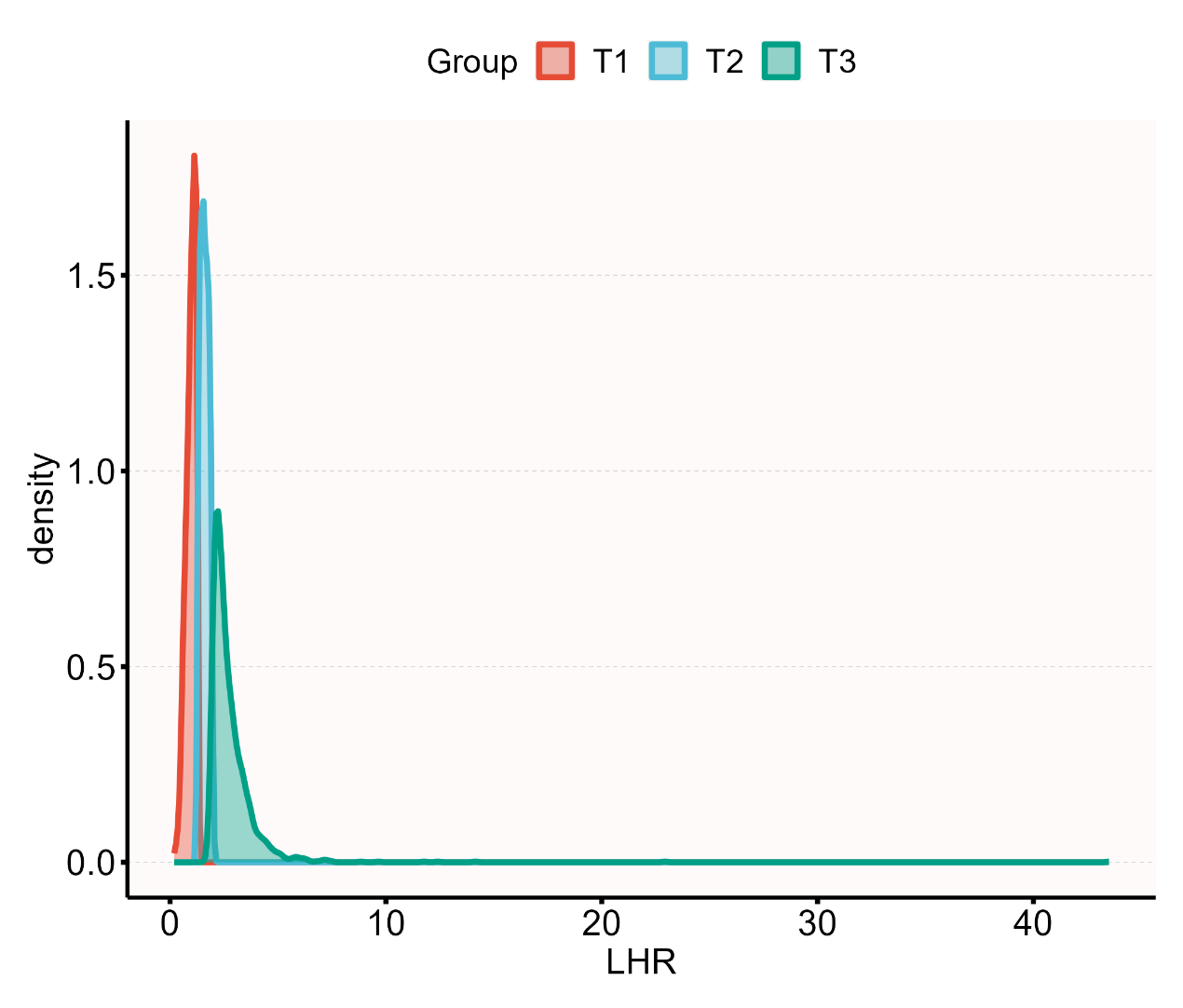


**Supplementary Figure 1** Density Plot of LHR Tertile Groups

T1, T2, and T3 represent the LHR tertile groups (T1: LHR ≤ 1.29, T2: 1.29 -1.94, T3: LHR ≥ 1.94). The density curves were constructed based on the LHR values of 5,323 participants.

Supplementary Table 2. Baseline characteristics according to all-cause mortality

| **Variable** | **All-cause mortality** | | | |
| --- | --- | --- | --- | --- |
|  | **Total(5323)** | **Alive (4599)** | **Death (724)** | ***P* value** |
| Gender, n (%) |  |  |  | 0.003 |
| Male | 2280 (42.83) | 1933 (42.03) | 347 (47.93) |  |
| Female | 3043 (57.17) | 2666 (57.97) | 377 (52.07) |  |
| Age(years) | 47.79 ± 17.64 | 44.90 ± 16.39 | 66.17 ± 13.80 | <0.001 |
| Race, n (%) |  |  |  | <0.001 |
| Non-Hispanic White | 2656 (49.90) | 2214 (48.14) | 442 (61.05) |  |
| Non-Hispanic Black | 1235 (23.20) | 1081 (23.51) | 154 (21.27) |  |
| Mexican American | 526 (9.88) | 473 (10.28) | 53 (7.32) |  |
| Other | 906 (17.02) | 831 (18.07) | 75 (10.36) |  |
| Education level, n (%) |  |  |  | <0.001 |
| Below high school | 426 (8.00) | 286 (6.22) | 140 (19.34) |  |
| High school | 1937 (36.39) | 1637 (35.59) | 300 (41.44) |  |
| Above high school | 2960 (55.61) | 2676 (58.19) | 284 (39.23) |  |
| Marital Status, n (%) |  |  |  | <0.001 |
| Married or living with partners | 2898 (54.44) | 2549 (55.43) | 349 (48.2) |  |
| Living alone | 2425 (45.56) | 2050 (44.57) | 375 (51.8) |  |
| Smoking status, n (%) |  |  |  | <0.001 |
| Current | 1300 (24.42) | 1097 (23.85) | 203 (28.04) |  |
| Former | 1424 (26.75) | 1125 (24.46) | 299 (41.3) |  |
| Never | 2599 (48.83) | 2377 (51.69) | 222 (30.66) |  |
| Alcohol use, n (%) |  |  |  | <0.001 |
| No | 1326 (24.91) | 1079 (23.46) | 247 (34.12) |  |
| Yes | 3997 (75.09) | 3520 (76.54) | 477 (65.88) |  |
| Hypertension, n (%) |  |  |  | <0.001 |
| No | 3460 (65.00) | 3177 (69.08) | 283 (39.09) |  |
| Yes | 1863 (35.00) | 1422 (30.92) | 441 (60.91) |  |
| Diabetes, n (%) |  |  |  | <0.001 |
| No | 4516 (84.84) | 3991 (86.78) | 525 (72.51) |  |
| Yes | 807 (15.16) | 608 (13.22) | 199 (27.49) |  |
| Prescribed medications, n (%) |  |  |  | <0.001 |
| No | 1572 (29.53) | 1516 (32.96) | 56 (7.73) |  |
| Yes | 3751 (70.47) | 3083 (67.04) | 668 (92.27) |  |
| ASCVD, n (%) |  |  |  | <0.001 |
| No | 4588 (86.19) | 4106 (89.28) | 482 (66.57) |  |
| Yes | 735 (13.81) | 493 (10.72) | 242 (33.43) |  |
| PIR | 1.94 (1.02, 3.94) | 2.06 (1.04, 4.14) | 1.49 (0.92, 2.77) | <0.001 |
| BMI, (kg/m^2^) | 30.52 ± 8.03 | 30.55 ± 8.05 | 30.27 ± 7.96 | 0.370 |
| Eosinophils, ×10^9^/L | 0.24 ± 0.18 | 0.24 ± 0.18 | 0.25 ± 0.22 | 0.023 |
| PA-MET | 600.00(0.00,2640.00) | 720.00(14.00,3120.00) | 28.00(0.00,840.00) | <0.001 |
| LHR | 1.59 (1.15, 2.18) | 1.61 (1.18, 2.20) | 1.42 (1.01, 2.03) | <0.001 |
| Follow up time | 106.95 ± 63.27 | 110.98 ± 63.46 | 81.34 ± 55.62 | <0.001 |

Data are presented as mean (SD) for continuous variables and percent for categorical variables. This table is based on the complete-case sample (n = 5323).

**Abbreviations:** LHR, lymphocyte-to-high-density lipoprotein cholesterol ratio; PIR, Ratio of family income to poverty; ASCVD, Atherosclerotic Cardiovascular Disease; BMI, body mass index; PA-MET physical activity metabolic equivalent; CVD, Cardiovascular Disease; CLRD, Chronic Lower Respiratory Disease;

Supplementary Table 3. Baseline characteristics according to CVD mortality

| **Variable** | **CVD mortality** | | | |
| --- | --- | --- | --- | --- |
|  | **Total(5323)** | **Alive (5140)** | **Death (183)** | ***P* value** |
| Gender, n (%) |  |  |  | 0.005 |
| Male | 2280 (42.83) | 2183 (42.47) | 97 (53.01) |  |
| Female | 3043 (57.17) | 2957 (57.53) | 86 (46.99) |  |
| Age(years) | 47.79 ± 17.64 | 47.08 ± 17.35 | 67.68 ± 13.60 | <0.001 |
| Race, n (%) |  |  |  | 0.005 |
| Non-Hispanic White | 2656 (49.90) | 2545 (49.51) | 111 (60.66) |  |
| Non-Hispanic Black | 1235 (23.20) | 1192 (23.19) | 43 (23.5) |  |
| Mexican American | 526 (9.88) | 516 (10.04) | 10 (5.46) |  |
| Other | 906 (17.02) | 887 (17.26) | 19 (10.38) |  |
| Education level, n (%) |  |  |  | <0.001 |
| Below high school | 426 (8.00) | 386 (7.51) | 40 (21.86) |  |
| High school | 1937 (36.39) | 1871 (36.4) | 66 (36.07) |  |
| Above high school | 2960 (55.61) | 2883 (56.09) | 77 (42.08) |  |
| Marital Status, n (%) |  |  |  | 0.008 |
| Married or living with partners | 2898 (54.44) | 2816 (54.79) | 82 (44.81) |  |
| Living alone | 2425 (45.56) | 2324 (45.21) | 101 (55.19) |  |
| Smoking status, n (%) |  |  |  | <0.001 |
| Current | 1300 (24.42) | 1249 (24.3) | 51 (27.87) |  |
| Former | 1424 (26.75) | 1354 (26.34) | 70 (38.25) |  |
| Never | 2599 (48.83) | 2537 (49.36) | 62 (33.88) |  |
| Alcohol use, n (%) |  |  |  | 0.004 |
| No | 1326 (24.91) | 1264 (24.59) | 62 (33.88) |  |
| Yes | 3997 (75.09) | 3876 (75.41) | 121 (66.12) |  |
| Hypertension, n (%) |  |  |  | <0.001 |
| No | 3460 (65.00) | 3389 (65.93) | 71 (38.8) |  |
| Yes | 1863 (35.00) | 1751 (34.07) | 112 (61.2) |  |
| Diabetes, n (%) |  |  |  | <0.001 |
| No | 4516 (84.84) | 4387 (85.35) | 129 (70.49) |  |
| Yes | 807 (15.16) | 753 (14.65) | 54 (29.51) |  |
| Prescribed medications, n (%) |  |  |  | <0.001 |
| No | 1572 (29.53) | 1562 (30.39) | 10 (5.46) |  |
| Yes | 3751 (70.47) | 3578 (69.61) | 173 (94.54) |  |
| ASCVD, n (%) |  |  |  | <0.001 |
| No | 4588 (86.19) | 4484 (87.24) | 104 (56.83) |  |
| Yes | 735 (13.81) | 656 (12.76) | 79 (43.17) |  |
| PIR | 1.94 (1.02, 3.94) | 1.98 (1.03, 3.98) | 1.37 (0.91, 2.71) | <0.001 |
| BMI, (kg/m^2^) | 30.52 ± 8.03 | 30.50 ± 8.01 | 30.99 ± 8.69 | 0.413 |
| Eosinophils, ×10^9^/L | 0.24 ± 0.18 | 0.24 ± 0.18 | 0.27 ± 0.21 | 0.008 |
| PA-MET | 600.00(0.00,2640.00) | 600.00(0.00,2817.85) | 18.67(0.00,870.00) | <0.001 |
| LHR | 1.59 (1.15, 2.18) | 1.60 (1.16, 2.19) | 1.36 (1.02, 1.93) | 0.001 |
| Follow up time | 106.95 ± 63.27 | 108.08 ± 63.34 | 75.37 ± 52.26 | <0.001 |

Data are presented as mean (SD) for continuous variables and percent for categorical variables. This table is based on the complete-case sample (n = 5323).

**Abbreviations:** LHR, lymphocyte-to-high-density lipoprotein cholesterol ratio; PIR, Ratio of family income to poverty; ASCVD, Atherosclerotic Cardiovascular Disease; BMI, body mass index; PA-MET physical activity metabolic equivalent; CVD, Cardiovascular Disease; CLRD, Chronic Lower Respiratory Disease;

Supplementary Table 4. Baseline characteristics according to CLRD mortality

| **Variable** | **CLRD mortality** | | | |
| --- | --- | --- | --- | --- |
|  | **Total(5323)** | **Alive (5232)** | **Death (91)** | ***P* value** |
| Gender, n (%) |  |  |  | 0.666 |
| Male | 2280 (42.83) | 2239 (42.79) | 41 (45.05) |  |
| Female | 3043 (57.17) | 2993 (57.21) | 50 (54.95) |  |
| Age(years) | 47.79 ± 17.64 | 47.44 ± 17.54 | 67.60 ± 10.65 | <0.001 |
| Race, n (%) |  |  |  | 0.004 |
| Non-Hispanic White | 2656 (49.90) | 2595 (49.6) | 61 (67.03) |  |
| Non-Hispanic Black | 1235 (23.20) | 1218 (23.28) | 17 (18.68) |  |
| Mexican American | 526 (9.88) | 524 (10.02) | 2 (2.2) |  |
| Other | 906 (17.02) | 895 (17.11) | 11 (12.09) |  |
| Education level, n (%) |  |  |  | <0.001 |
| Below high school | 426 (8.00) | 406 (7.76) | 20 (21.98) |  |
| High school | 1937 (36.39) | 1891 (36.14) | 46 (50.55) |  |
| Above high school | 2960 (55.61) | 2935 (56.1) | 25 (27.47) |  |
| Marital Status, n (%) |  |  |  | 0.743 |
| Married or living with partners | 2898 (54.44) | 2850 (54.47) | 48 (52.75) |  |
| Living alone | 2425 (45.56) | 2382 (45.53) | 43 (47.25) |  |
| Smoking status, n (%) |  |  |  | <0.001 |
| Current | 1300 (24.42) | 1272 (24.31) | 28 (30.77) |  |
| Former | 1424 (26.75) | 1371 (26.2) | 53 (58.24) |  |
| Never | 2599 (48.83) | 2589 (49.48) | 10 (10.99) |  |
| Alcohol use, n (%) |  |  |  | 0.042 |
| No | 1326 (24.91) | 1295 (24.75) | 31 (34.07) |  |
| Yes | 3997 (75.09) | 3937 (75.25) | 60 (65.93) |  |
| Hypertension, n (%) |  |  |  | <0.001 |
| No | 3460 (65.00) | 3426 (65.48) | 34 (37.36) |  |
| Yes | 1863 (35.00) | 1806 (34.52) | 57 (62.64) |  |
| Diabetes, n (%) |  |  |  | 0.003 |
| No | 4516 (84.84) | 4449 (85.03) | 67 (73.63) |  |
| Yes | 807 (15.16) | 783 (14.97) | 24 (26.37) |  |
| Prescribed medications, n (%) |  |  |  | <0.001 |
| No | 1572 (29.53) | 1569 (29.99) | 3 (3.3) |  |
| Yes | 3751 (70.47) | 3663 (70.01) | 88 (96.7) |  |
| ASCVD, n (%) |  |  |  | <0.001 |
| No | 4588 (86.19) | 4522 (86.43) | 66 (72.53) |  |
| Yes | 735 (13.81) | 710 (13.57) | 25 (27.47) |  |
| PIR | 1.94 (1.02, 3.94) | 1.96 (1.02, 3.97) | 1.37 (0.98, 2.62) | 0.017 |
| BMI, (kg/m^2^) | 30.52 ± 8.03 | 30.55 ± 8.04 | 28.71 ± 7.74 | 0.030 |
| Eosinophils, ×10^9^/L | 0.24 ± 0.18 | 0.24 ± 0.18 | 0.29 ± 0.30 | 0.008 |
| PA-MET | 600.00(0.00,2640.00) | 600.00(0.00,2641.50) | 0.00(0.00,476.25) | < 0.001 |
| LHR | 1.59 (1.15, 2.18) | 1.59 (1.16, 2.19) | 1.26 (0.83, 2.03) | <0.001 |
| Follow up time | 106.95 ± 63.27 | 107.34 ± 63.28 | 84.42 ± 58.68 | <0.001 |

Data are presented as mean (SD) for continuous variables and percent for categorical variables. This table is based on the complete-case sample (n = 5323).

**Abbreviations:** LHR, lymphocyte-to-high-density lipoprotein cholesterol ratio; PIR, Ratio of family income to poverty; ASCVD, Atherosclerotic Cardiovascular Disease; BMI, body mass index; PA-MET physical activity metabolic equivalent; CVD, Cardiovascular Disease; CLRD, Chronic Lower Respiratory Disease;


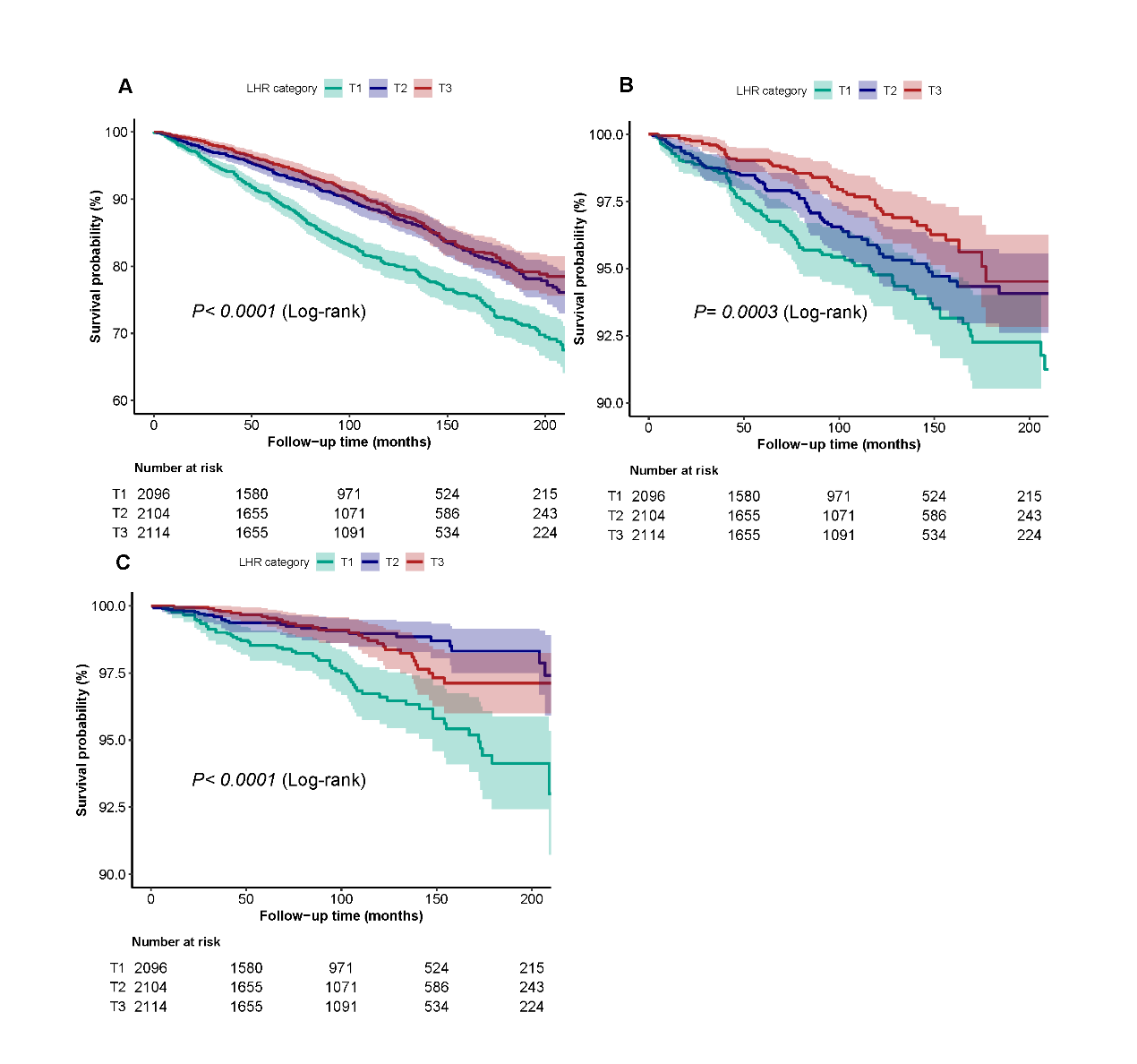


**Supplementary Figure 2** Kaplan–Meier Curves for All-Cause (**A**), CVD(**B**), and CLRD Mortality (**C)** by LHR Tertiles

T1, T2, and T3 represent LHR tertile groups. LHR: lymphocyte-to-high-density lipoprotein cholesterol ratio; CLRD: chronic lower respiratory disease.

**Supplementary Table 5.** Multivariate analysis of the association between LHR and all-cause, CVD, and CLRD mortality in asthma after complete-case analysis(**N=5323**)

| **LHR** | **All -cause mortality** | | | | | | |
| --- | --- | --- | --- | --- | --- | --- | --- |
|  | **Events (%)** | **Crude Model** |  | **Model 1** |  | **Model 2** |  |
|  |  | **HR (95%CI)** | **p-Value** | **HR (95%CI)** |  | **HR (95%CI)** | ***p-Value*** |
| LHR log_2_ | 724(13.6) | 0.70(0.63~0.78) | <0.001 | 0.84(0.75-0.94) | 0.002 | 0.81(0.73-0.91) | <0.001 |
| LHR category |  |  |  |  |  |  |  |
| T1(≤1.29) | 311(17.6) | 1(Ref) |  | 1(Ref) |  | 1(Ref) |  |
| T2(1.29-1.94) | 211(11.9) | 0.63(0.53~0.75) | <0.001 | 0.82(0.68~0.98) | 0.033 | 0.79(0.66~0.95) | 0.010 |
| T3(≥1.94) | 202(11.3) | 0.62(0.52~0.74) | <0.001 | 0.78(0.65~0.95) | 0.013 | 0.73(0.60~0.89) | 0.002 |
| *P* for trend |  |  | <0.001 |  | 0.010 |  | 0.001 |
|  | **CVD mortality** | | | | | | |
|  |  | **Crude Model** |  | **Model 1** |  | **Model 2** |  |
|  |  | **HR (95%CI)** | **p-Value** | **HR (95%CI)** |  | **HR (95%CI)** | ***p-Value*** |
| LHR log_2_ | 183(3.4) | 0.70(0.57~0.87) | 0.001 | 0.83(0.67~1.03) | 0.096 | 0.79(0.63~0.99) | 0.039 |
| LHR category |  |  |  |  |  |  |  |
| T1(≤1.29) | 80(4.5) | 1(Ref) |  | 1(Ref) |  | 1(Ref) |  |
| T2(1.29-1.94) | 58(3.3) | 0.68(0.48~0.95) | 0.024 | 0.91(0.64~1.29) | 0.595 | 0.86(0.60~1.22) | 0.399 |
| T3(≥1.94) | 45(2.5) | 0.53(0.37~0.77) | 0.001 | 0.66(0.45~0.98) | 0.041 | 0.60(0.40~0.90) | 0.013 |
| *P* for trend |  |  | 0.001 |  | 0.045 |  | 0.014 |
|  | **CLRD mortality** | | | | | | |
|  |  | **Crude Model** |  | **Model 1** |  | **Model 2** |  |
|  |  | **HR (95%CI)** | **p-Value** | **HR (95%CI)** |  | **HR (95%CI)** | ***p-Value*** |
| LHR log_2_ | 91(1.7) | 0.49(0.36~0.65) | <0.001 | 0.58(0.43~0.79) | 0.001 | 0.58(0.43~0.80) | 0.001 |
| LHR category |  |  |  |  |  |  |  |
| T1(≤1.29) | 47(2.7) | 1(Ref) |  | 1(Ref) |  | 1(Ref) |  |
| T2(1.29-1.94) | 19(1.1) | 0.38(0.22~0.64) | <0.001 | 0.47(0.27~0.82) | 0.007 | 0.47(0.27~0.82) | 0.008 |
| T3(≥1.94) | 25(1.4) | 0.50(0.31~0.82) | 0.006 | 0.64(0.38~1.08) | 0.095 | 0.62(0.36~1.06) | 0.079 |
| *P* for trend |  |  | 0.003 |  | 0.058 |  | 0.048 |

**Notes:**

**Crude Model**, unadjusted;

**Model 1:** Adjusted for gender, age, race, education level, marital status, PIR, BMI, Eosinophils, alcohol use, smoking status;

**Model 2:** Further adjusted for ASCVD, hypertension, diabetes, Prescribed medications, PA-MET;

**Abbreviations:** LHR lymphocyte-to-high-density lipoprotein cholesterol ratio; CVD: Cardiovascular Disease; PIR: Ratio of family income to poverty; ASCVD: Atherosclerotic Cardiovascular Disease; BMI body mass index; PA-MET physical activity metabolic equivalent; Ref：Reference; CLRD: Chronic Lower Respiratory Disease;

**Supplementary Table 6.** Multivariate analysis of the association between LHR and all-cause, CVD, and CLRD mortality in asthma after removing outliers beyond 3 standard deviations (**N=6274**)

| **LHR** | **All -cause mortality** | | | | | | |
| --- | --- | --- | --- | --- | --- | --- | --- |
|  | **Events (%)** | **Crude Model** |  | **Model 1** |  | **Model 2** |  |
|  |  | **HR (95%CI)** | **p-Value** | **HR (95%CI)** | **p-Value** | **HR (95%CI)** | **p-Value** |
| LHR log_2_ | 895(14.3) | 0.65(0.59~0.71) | <0.001 | 0.82(0.74-0.90) | <0.001 | 0.79(0.72-0.88) | <0.001 |
| LHR category |  |  |  |  |  |  |  |
| T1(≤1.29) | 390(18.6) | 1(Ref) |  | 1(Ref) |  | 1(Ref) |  |
| T2(1.29-1.94) | 270(12.8) | 0.65(0.55~0.75) | <0.001 | 0.86(0.73~1.01) | 0.071 | 0.83(0.71~0.98) | 0.027 |
| T3(≥1.94) | 235(11.3) | 0.58(0.49~0.68) | <0.001 | 0.81(0.68~0.96) | 0.017 | 0.76(0.64~0.91) | 0.002 |
| *P* for trend |  |  | <0.001 |  | 0.014 |  | 0.002 |
|  | **CVD mortality** | | | | | | |
|  |  | **Crude Model** |  | **Model 1** |  | **Model 2** |  |
|  |  | **HR (95%CI)** | **p-Value** | **HR (95%CI)** | **p-Value** | **HR (95%CI)** | **p-Value** |
| LHR log_2_ | 226(3.6) | 0.64(0.53~0.77) | <0.001 | 0.79(0.65~0.97) | 0.023 | 0.75(0.62~0.92) | 0.006 |
| LHR category |  |  |  |  |  |  |  |
| T1(≤1.29) | 99(4.7) | 1(Ref) |  | 1(Ref) |  | 1(Ref) |  |
| T2(1.29-1.94) | 76(3.6) | 0.72(0.53~0.97) | 0.031 | 0.99(0.73~1.35) | 0.962 | 0.94(0.69~1.28) | 0.678 |
| T3(≥1.94) | 51(2.5) | 0.49(0.35~0.69) | <0.001 | 0.69(0.48~0.99) | 0.041 | 0.61(0.42~0.89) | 0.009 |
| *P* for trend |  |  | <0.001 |  | 0.055 |  | 0.012 |
|  | **CLRD mortality** | | | | | | |
|  |  | **Crude Model** |  | **Model 1** |  | **Model 2** |  |
|  |  | **HR (95%CI)** | **p-Value** | **HR (95%CI)** | **p-Value** | **HR (95%CI)** | **p-Value** |
| LHR log_2_ | 114(1.8) | 0.44(0.34~0.57) | <0.001 | 0.56(0.42~0.74) | <0.001 | 0.56(0.42~0.74) | <0.001 |
| LHR category |  |  |  |  |  |  |  |
| T1(≤1.29) | 62(3.0) | 1(Ref) |  | 1(Ref) |  | 1(Ref) |  |
| T2(1.29-1.94) | 23(1.1) | 0.34(0.21~0.56) | <0.001 | 0.45(0.28~0.74) | 0.002 | 0.44(0.27~0.73) | 0.001 |
| T3(≥1.94) | 29(1.4) | 0.45(0.29~0.69) | <0.001 | 0.66(0.41~1.07) | 0.095 | 0.65(0.39~1.06) | 0.083 |
| *P* for trend |  |  | <0.001 |  | 0.039 |  | 0.032 |

**Notes:**

**Crude Model**, unadjusted;

**Model 1:** Adjusted for gender, age, race, education level, marital status, PIR, BMI, Eosinophils, alcohol use, smoking status;

**Model 2:** Further adjusted for ASCVD, hypertension, diabetes, Prescribed medications, PA-MET;

**Abbreviations:** LHR lymphocyte-to-high-density lipoprotein cholesterol ratio; CVD: Cardiovascular Disease; PIR: Ratio of family income to poverty; ASCVD: Atherosclerotic Cardiovascular Disease; BMI body mass index; PA-MET physical activity metabolic equivalent; Ref：Reference; CLRD: Chronic Lower Respiratory Disease;

**Supplementary Table 7.** Association between LHR and all-cause, CVD, and CLRD mortality with E-values for robustness evaluation(**N=6314**)

| **LHR** | **All -cause mortality** | | | | | | |
| --- | --- | --- | --- | --- | --- | --- | --- |
|  | **Events (%)** | **Crude Model** |  | **Model 1** |  | **Model 2** |  |
|  |  | **HR (95%CI)** | **E-Value** | **HR (95%CI)** | **E-Value** | **HR (95%CI)** | ***E-Value*** |
| LHR log_2_ | 905(14.3) | 0.68(0.62~0.75) | **2.30** | 0.85(0.77-0.94) | **1.63** | 0.82(0.74-0.91) | **1.74** |
| LHR category |  |  |  |  |  |  |  |
| T1(≤1.29) | 390(18.6) | 1(Ref) |  | 1(Ref) |  | 1(Ref) |  |
| T2(1.29-1.94) | 270(12.8) | 0.65(0.55~0.75) | **2.45** | 0.86(0.73~1.01) | **1.60** | 0.83(0.71~0.98) | **1.70** |
| T3(≥1.94) | 245(11.6) | 0.59(0.50~0.69) | **2.78** | 0.82(0.69~0.98) | **1.74** | 0.77(0.65~0.92) | **1.92** |
|  | **CVD mortality** | | | | | | |
|  |  | **Crude Model** |  | **Model 1** |  | **Model 2** |  |
|  |  | **HR (95%CI)** | **E-Value** | **HR (95%CI)** | **E-Value** | **HR (95%CI)** | ***E-Value*** |
| LHR log_2_ | 229(3.6) | 0.67(0.56~0.81) | **2.35** | 0.83(0.68~1.01) | **1.70** | 0.79(0.65~0.96) | **1.85** |
| LHR category |  |  |  |  |  |  |  |
| T1(≤1.29) | 99(4.7) | 1(Ref) |  | 1(Ref) |  | 1(Ref) |  |
| T2(1.29-1.94) | 76(3.6) | 0.72(0.53~0.97) | **2.12** | 0.99(0.72~1.35) | **1.36** | 0.94(0.69~1.28) | **1.32** |
| T3(≥1.94) | 54(2.6) | 0.51(0.37~0.72) | **3.33** | 0.70(0.49~1.00) | **2.35** | 0.63(0.44~0.91) | **2.55** |
|  | **CLRD mortality** | | | | | | |
|  |  | **Crude Model** |  | **Model 1** |  | **Model 2** |  |
|  |  | **HR (95%CI)** | **E-Value** | **HR (95%CI)** | **E-Value** | **HR (95%CI)** | **E-Value** |
| LHR log_2_ | 115(1.8) | 0.46(0.35~0.60) | **3.77** | 0.59(0.45~0.77) | **2.78** | 0.59(0.45~0.77) | **2.84** |
| LHR category |  |  |  |  |  |  |  |
| T1(≤1.29) | 62(3.0) | 1(Ref) |  | 1(Ref) |  | 1(Ref) |  |
| T2(1.29-1.94) | 23(1.1) | 0.34(0.21~0.56) | **5.33** | 0.45(0.28~0.74) | **3.87** | 0.45(0.27~0.73) | **3.87** |
| T3(≥1.94) | 30(1.4) | 0.45(0.29~0.70) | **3.87** | 0.68(0.42~1.09) | **2.30** | 0.66(0.41~1.08) | **2.40** |

**Notes:**

**Crude Model**, unadjusted;

**Model 1:** Adjusted for gender, age, race, education level, marital status, PIR, BMI, Eosinophils, alcohol use, smoking status;

**Model 2:** Further adjusted for ASCVD, hypertension, diabetes, Prescribed medications, PA-MET;

**Abbreviations:** LHR lymphocyte-to-high-density lipoprotein cholesterol ratio; CVD: Cardiovascular Disease; PIR: Ratio of family income to poverty; ASCVD: Atherosclerotic Cardiovascular Disease; BMI body mass index; PA-MET physical activity metabolic equivalent; Ref：Reference; CLRD: Chronic Lower Respiratory Disease;


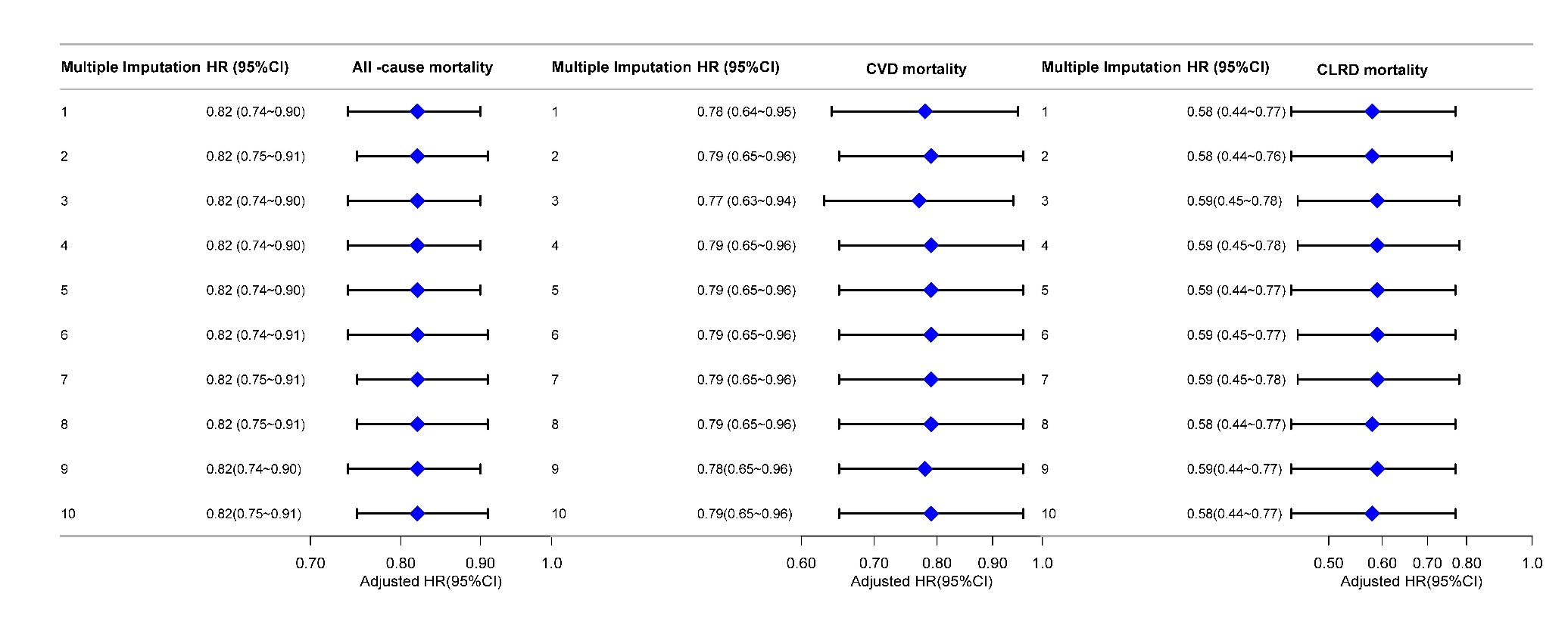


**Supplementary Figure 3**. Results for Multiple Imputations (10 Times) for All-Cause Mortality, CVD Mortality, and CLRD Mortality

Hazard ratios (HRs) and 95% confidence intervals (CIs) were calculated using multiple imputations (10 datasets) to assess the association between log_2_LHR and all-cause mortality, CVD mortality, and CLRD mortality.


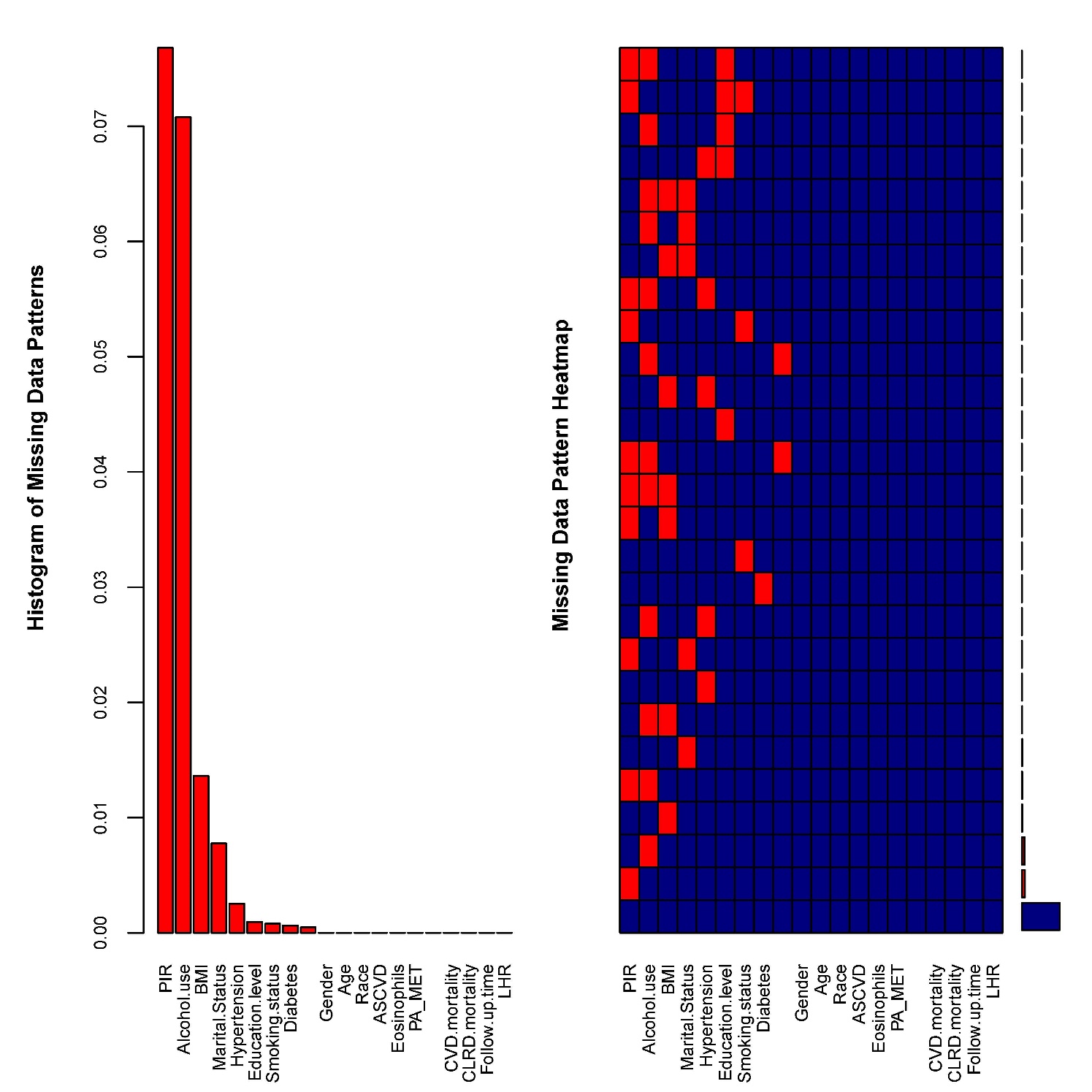


**Supplementary Figure 4** Distribution and Proportions of Missing Values Across Variables

**Caption:** The bar plot on the left shows the proportion of missing values for each variable, with PIR (Poverty Income Ratio) having the highest proportion (**7.68%**), followed by Alcohol Use (**7.08%**) and BMI (**1.36%**). The heatmap on the right illustrates the missing data patterns, where red indicates missing values and blue indicates observed values. Overall, the proportion of missing values is low, and the distribution shows no significant bias.
